# HDAC1/2 control mesothelium/ovarian cancer adhesive interactions impacting on Talin-1-α5β1-integrin-mediated actin cytoskeleton and extracellular matrix protein remodeling

**DOI:** 10.1186/s13046-023-02930-8

**Published:** 2024-01-23

**Authors:** Michela Terri, Pilar Sandoval, Giulio Bontempi, Claudia Montaldo, Henar Tomero-Sanz, Valeria de Turris, Flavia Trionfetti, Lucía Pascual-Antón, Irene Clares-Pedrero, Cecilia Battistelli, Sergio Valente, Clemens Zwergel, Antonello Mai, Laura Rosanò, Miguel Ángel del Pozo, Miguel Sánchez-Álvarez, Carlos Cabañas, Marco Tripodi, Manuel López-Cabrera, Raffaele Strippoli

**Affiliations:** 1 Department of Molecular Medicine, Sapienza University of Rome, Viale Regina Elena 324, 00161 Rome, Italy; 2 National Institute for Infectious Diseases L. Spallanzani, IRCCS, Via Portuense, 292, 00149 Rome, Italy; 3 Tissue and Organ Homeostasis Program, Cell-Cell Communication and Inflammation Unit, Centro de Biología Molecular “Severo Ochoa” (UAM-CSIC), Consejo Superior de Investigaciones Científicas, 28049 Madrid, Spain; 4 Center for Life Nano- & Neuro-Science, Istituto Italiano di Tecnologia (IIT), 00161 Rome, Italy; 5 Department of Immunology, Ophthalmology and Otorhinolaryngology, School of Medicine, Universidad Complutense de Madrid, 28040 Madrid, Spain; 6 Department of Drug Chemistry and Technologies, Laboratory affiliated to Istituto Pasteur Italia-Fondazione Cenci Bolognetti, Sapienza University of Rome, Rome, Italy; 7 Institute of Molecular Biology and Pathology, CNR, Rome, Italy; 8 Mechanoadaptation and Caveolae Biology Lab, Area of Cell and Developmental Biology, Centro Nacional de Investigaciones Cardiovasculares (CNIC), Madrid, Spain; 9 Cell Compartmentalization, Homeostasis and Inflammation lab. Department of Metabolic and Immunity Diseases, Instituto de Investigaciones Biomédicas “Sols-Morreale”, 28029 Madrid, Spain

**Keywords:** Peritoneum, Peritoneal Carcinomatosis, Epithelial ovarian Cancer, HDAC1–2, MS-275, Mesothelial to mesenchymal transition (MMT), Extracellular matrix, Integrin, Talin1, Fibronectin-1, Actin cytoskeleton

## Abstract

**Background:**

Peritoneal metastasis, which accounts for 85% of all epithelial ovarian carcinoma (EOC) metastases, is a multistep process that requires the establishment of adhesive interactions between cancer cells and the peritoneal membrane. Interrelations between EOC and the mesothelial stroma are critical to facilitate the metastatic process. No data is available so far on the impact of histone acetylation/deacetylation, a potentially relevant mechanism governing EOC metastasis, on mesothelial cells (MCs)-mediated adhesion.

**Methods:**

Static adhesion and peritoneal clearance experiments were performed pretreating mesenchymal-like MCs and platinum—sensitive/resistant EOC cell lines with MS-275—a Histone deacetylase (HDAC)1–3 pharmacological inhibitor currently used in combination trials. Results were acquired by confocal microscopy and were analyzed with an automated Opera software.

The role of HDAC1/2 was validated by genetic silencing. The role of α4-, α5-α1 Integrins and Fibronectin-1 was validated using specific monoclonal antibodies.

Quantitative proteomic analysis was performed on primary MCs pretreated with MS-275. Decellularized matrices were generated from either MS-275-exposed or untreated cells to study Fibronectin-1 extracellular secretion. The effect of MS-275 on β1 integrin activity was assessed using specific monoclonal antibodies. The role of Talin-1 in MCs/EOC adhesion was analyzed by genetic silencing. Talin-1 ectopic expression was validated as a rescue tool from MS-275-induced phenotype. The in vivo effect of MS-275-induced MC remodeling was validated in a mouse model of peritoneal EOC dissemination.

**Results:**

Treatment of MCs with non-cytotoxic concentrations of MS-275 caused a consistent reduction of EOC adhesion. Proteomic analysis revealed several pathways altered upon MC treatment with MS-275, including ECM deposition/remodeling, adhesion receptors and actin cytoskeleton regulators. HDAC1/2 inhibition hampered actin cytoskeleton polymerization by downregulating actin regulators including Talin-1, impairing β1 integrin activation, and leading to abnormal extracellular secretion and distribution of Fibronectin-1. Talin-1 ectopic expression rescued EOC adhesion to MS-275-treated MCs. In an experimental mouse model of metastatic EOC, MS-275 limited tumor invasion, Fibronectin-1 secretion and the sub-mesothelial accumulation of MC-derived carcinoma-associated fibroblasts.

**Conclusion:**

Our study unveils a direct impact of HDAC-1/2 in the regulation of MC/EOC adhesion and highlights the regulation of MC plasticity by epigenetic inhibition as a potential target for therapeutic intervention in EOC peritoneal metastasis.

**Supplementary Information:**

The online version contains supplementary material available at 10.1186/s13046-023-02930-8.

## Introduction

Ovarian cancer is the fifth leading cause of cancer-related deaths among women, and the second one among gynecologic cancers [1]. The standard regimen for advanced epithelial carcinoma (EOC) is debulking surgery following cis-platinum/taxane-based chemotherapy. During treatment, resistance often develops leading to relapse and therapeutic failure. Approximately 75% of patients with advanced stages will eventually experience recurrence [2], and almost all patients with recurrent disease ultimately develop cis-platinum resistance, with poor prognosis and only 40% of patients surviving for 5 years [3, 4]. Improved treatment options are urgently needed.

EOC most common and earliest route of metastasis is the so-called transcoelomic route. EOC cells from primary tumors protrude to the peritoneal surface and detach as single cells or clusters, and they then disseminate to the peritoneum surface through a passive mechanism [5]. EOC spread increases the filtration rate to the peritoneal cavity due to the increased microvessels in the membrane-surface lining the peritoneal cavity and creates obstruction in the lymphatic system, causing an accumulation of fluid in peritoneal cavities, called malignant ascites [6].

In accordance with the “seed and soil” Paget’s theory, it was demonstrated that solid tumors prepare their pre-metastatic niche through the secretion of various stimuli such as cytokines, chemokines and other extracellular mediators [7–9].

While healthy mesothelium is repulsive to EOC cell adhesion, this process is facilitated by pathological modification of mesothelial surfaces.

In response to EOC-driven stimuli, mesothelial cells (MCs) undergo MMT (mesothelial to mesenchymal transition) acquiring a fibroblast-like phenotype with invasive properties and constitute the main component of the Cancer-Associated Fibroblasts (CAFs) population [10, 11].

Integrins act as a bridge between the ECM and the Actin cytoskeleton inside the cell to form integrin adhesion complexes [12]. Binding to Talin-1 is the key step triggering integrin adhesion and many integrin-mediated functions [13].

Direct interactions between EOC cells and MCs are principally mediated by β1-Integrins, in which the β1-Integrin subunit can heterodimerize with many different α Integrin subunits (including -α2, α4, αv, α5, α6). β1 Integrins play a role in ECM remodeling and in the formation of spheroids, three-dimensional cellular aggregates found in cancer patients and used as an experimental model of micrometastasis formation [14]. In particular, the key role of α4β1 and α5β1 Integrins in the first stage of the adhesion process has been demonstrated using different experimental approaches [10, 14, 15]. As a part of the EMT process in EOC, E-Cadherin downregulation leads to upregulation and activation of α5β1 Integrin, which facilitates EOC cell adhesion to mesothelium [16]. Treatment with inhibiting antibodies against β1-Integrin partially blocks EOC adhesion to MCs [17]. While plenty of information is already available on EOC Integrins, the role of MC Integrins in the same process still needs to be fully elucidated.

Histone acetylation and deacetylation play an essential role in modifying chromatin structure and in regulating gene expression in eukaryotic cells [18, 19]. Key enzymes, that modify histone proteins and thereby regulate gene expression, are histone acetyltransferases (HATs) and histone deacetylases (HDACs). In mammals, both these acetylating/deacetylating enzymes are components of multiprotein complexes containing other proteins known to exert their role in transcriptional activation/repression. To date, 18 distinct human HDACs have been reported, grouped into four classes (I-IV).

Epigenetic mechanisms are implicated in tumorigenesis. Indeed, histone deacetylases have crucial roles in cancer cells where they are often overexpressed, increasing proliferation, and causing cell de-differentiation [19–21].

In the last years, many epigenetic inhibitors have been designed and are currently being validated especially in the therapy of tumors and nontumoral fibrotic pathologies [22]. In addition to pan-HDAC inhibitors such as trichostatin A and vorinostat, small molecules have been designed to selectively inhibit the activity of specific HDAC classes/isoforms [23].

Besides tumorigenesis, histone deacetylases impact cellular plasticity in non-transformed cellular systems. In a previous study, we demonstrated that the inhibition of HDAC1–3 with MS-275 (a class I pharmacological inhibitor) in MCs derived from peritoneal dialysis (PD) patients promotes the re-acquisition of an epithelial-like morphology and the reduction of their invasive abilities [24].

The aim of this study is the analysis of epigenetic mechanisms regulating MC/EOC adhesion. Through the use of specific class I pharmacological inhibitors and specific genetic silencing, we revealed the role of HDAC1–2 in controlling α5β1 Integrin-mediated EOC cell adhesion to MCs.

Of note, MS-275 was demonstrated to perturb the expression of actin-interacting proteins such as Talin-1, Zyxin and α-Actinin-1, resulting in changes in actin cytoskeleton polymerization, as well as FN-1 deposition and organization, with both processes leading to reduced α5β1 integrin activation and diminished EOC cell/MC adhesion. Mechanistically Talin-1 ectopic expression was demonstrated to rescue the impaired MC/EOC adhesion observed upon MS-275 treatment.

Accordingly, treatment with MS-275 in the peritoneal pre-metastatic niche in mice reduced tumor colonization, suggesting that class I HDAC-dependent effects could be crucial in peritoneal carcinomatosis. Together, these results indicate that bidirectional cross-talks between EOC cells and mesenchymal-like MC are crucial to form a suitable metastatic niche. We suggest the regulation of MC plasticity by class I HDAC pharmacological inhibition as a possible target for therapeutic intervention in EOC peritoneal metastasis.

## Materials and methods

### Patient biopsies

A total of 8 ovarian cancer peritoneal implant biopsies were evaluated. Control parietal peritoneal membrane biopsies were obtained from non-oncological related cases (*n* = 3). All tissue samples were fixed with neutral buffered formalin for 24 hours and processed routinely for immunohistochemical analysis. The study was carried out in accordance with Good Clinical Practice guidelines and applicable regulations, as well as the ethical principles that have their origin in the Declaration of Helsinki. All included patients had signed informed consent forms and the study was approved by the Clinical Ethics Committee of Fundación Jiménez Díaz – QuirónSalud (ethic approval number:11/17) (Madrid, Spain).

### Patients and cell cultures

Effluent-derived mesenchymal MCs were isolated from 9 patients performing peritoneal dialysis as described previously [25]. All included patients had signed informed consent forms and the study was approved by the Ethics Committee of Clinic Investigation of Sapienza University ref.: 4697_2017 (Roma, Italy). MCs from PD effluents express the mesothelial markers intercellular adhesion molecule (ICAM)-1 and Cytokeratins 8–18, although at lower levels than healthy HPMCs. MC cultures were negative for the endothelial marker CD31 and the pan-leukocyte marker CD45 [26–28]. Effluent-derived MCs were cultured in Earle’s M199 supplemented with 10% FBS (GIBCO® Life Technology, Monza, Italy) 2 mM L-glutamine (EuroClone), 100 U/ml penicillin, 100 μg/ml streptomycin and amphotericin B (2.5 μg/ml) (all from Gibco-Life Technologies). The human mesothelial cell line MeT-5A (ATCC, Rockville, MD) was cultured in Earle’s M199 as above (except for amphotericin B). This cell line was isolated from pleural fluids obtained from a non-cancerous individual. SKOV3 (ATCC, Rockville, MD) cells were cultured in McCoy’s medium (Sigma-Aldrich, Saint Louis, MO). SKOV3 cells stably expressing GFP were from Lopez-Cabrera laboratory.

OVCAR-3 (ATCC, Rockville, MD) cells were cultured in RPMI Medium (Sigma-Aldrich,). 293 T cells (ATCC) were cultured in Dulbecco’s modified Eagle’s medium (DMEM) from Sigma-Aldrich. Kuramochi cell line was provided by the National Institutes of Biomedical Innovation (NIBIOHN) Osaka, Japan, and was grown in RPMI (as above). All cell media were supplemented with 10% FBS, 2 mM L-glutamine, 100 U/ml penicillin, and 100 μg/ml streptomycin.

Immortalized cell lines and primary cells were grown at 37 °C, in a humidified atmosphere with 5% CO_2_. To induce the acquisition of mesenchymal-like features, Met-5A cells were treated with TGFβ1 (2 ng/ml) in combination with IL-1β (0.5 ng/ml).

MCs from PD patients and MET5A cells were treated with DMSO or MS-275 (250 nM). SKOV-3 and OVCAR-3 cells were treated with DMSO or MS-275 (respectively 2.5 μM and 1 μM).

### Stable cell transfection

OVCAR-3 cells stably expressing GFP were generated for functional studies. For the production of lentiviral particles, 293 T cells were co-transfected with 5 μg of lentiviral vectors pLenti-C-mGFP-P2A-Puro (Origene, PS100093), 5 μg of pMD2-VSV-G ENV, 2.5 μg of pRSV-Rev, 2.5 μg of pMDLg/pRRE by using the calcium phosphate method. After 48 hours, the supernatant containing lentiviral particles was recovered, ultracentrifuged at 19.800 rpm on an SW28 rotor for 2 hours, and resuspended in phosphate-buffered saline (PBS) (500 μl for 20 ml of supernatant). OVCAR-3 cells were infected with 80 μl of viral suspension in a medium supplemented with polybrene (4 μg/ml) for 8 hours. Two consecutive rounds of infections were performed to improve the efficiency.

### Antibodies and chemicals

The primary antibodies for western blotting experiments were: rabbit anti-TGFβ-REC1 (ABF17–1), mouse anti- FN (F7387) from Sigma-Aldrich (Saint Louis, MO), mouse anti-E-cadherin (BD610181) from BD-Transduction Laboratories (Franklin Lakes, NJ), mouse anti-HSP90A-HSP90B/Hsp90 03B1α/β (sc-13,119), mouse anti-TUBA/alpha-tubulin (sc-32,293) from Santa Cruz Biotechnology (Dallas, TX), mouse anti-Talin (TA205) from Thermo Fisher Scientific (Waltham, MA USA). HRP– conjugated secondary antibodies used were purchased from Jackson immune research (Philadelphia, PA, USA): anti-rabbit (JI 711–036-152), anti-mouse (JI 715–036-150).

Antibodies for Immunofluorescence experiments were rabbit anti-collagen (NB600–480) from Novus Biological, (Litterton, CO, USA); rabbit anti- FN (ab2413), mouse anti- Integrin β1 (ab30394) from Abcam, (Cambridge, UK) mouse anti-integrin β1(clone HUTS21), rat anti-Integrin β1 (clone 9EG7, 550,531) BD Pharmigen (Franklin Lakes, NJ, USA), Cy3-conjugated anti-rat secondary antibodies (Jackson ImmunoResearch, 112–165-003), anti-rabbit Alexa Fluor 488-conjugated (A21206), anti-mouse Alexa Fluor 488-conjugated (A32723), Alexa fluor Phalloidin 647 (A2228), Rhodamine Phalloidin (R415), Hoechst 33342 (H21492) from Thermo Fisher Scientific. Antibody used for inhibit targets: Mouse monoclonal (HP2/1) to Integrin α4/CD49d. Mouse monoclonal P1D6 to Integrin α5/CD49E.

Anti-Integrin β HUTS21 [29], anti-Integrin α4 HP2/1 [30], anti-Integrin α5 P1D6 [31] were from Prof. Cabañas laboratory. MS-275 and MC-3105 were from the Mai lab. IL-1β was from R&D system (201-LB-010/CF) and TGF-β1 (100-21C) was from PeproTech (Rocky Hill, NJ, USA). PKH26 red fluorescent cell linker kit for general cell membrane labeling (Cat#PKH26GL-1KT), was from Sigma-Aldrich).

### Western blotting

Monolayers of effluent-derived MCs or MeT-5A cells were lysed in CelLytic™ MT Cell Lysis Reagent (Sigma-Aldrich), and proteins were quantified by Bradford protein assay reagent (Biorad Hercules, CA).

Laemmli SDS sample buffer was added and samples were boiled for 5 minutes at 95 °C and were loaded on acrylamide gels. Gels were electrophoresed at 100 V in Running Buffer (25 mM Tris, 190 mM glycine; 0.1% SDS) and then transferred to a Nitrocellulose membrane in Transfer Buffer (50 mM Tris, 40 mM glycine; 0.1% SDS; 20% Methanol). Blots were blocked in 5% non-fat milk prepared in TBS-Tween (10 mM Tris-HCl pH 7.5; 150 mM NaCl; 0.05% Tween 20) and incubated overnight with the primary antibody. The day after the blots were incubated with HRP-conjugated species-specific secondary antibodies. Nitrocellulose-bound antibodies were detected by chemiluminescence with ECL (Immobilon Western HRP substrate, Millipore) Acquisition of blots was performed with Chemidoc Touch imaging system and analysed with Image Lab Software release 6.0 (Bio-Rad Laboratories). Molecular size marker ladder (#PM2610) were from Thermo Fisher Scientific.

### Reverse-transcriptase polymerase chain reaction

RNA, extracted from cell cultures with RNeasy Mini Kit (Qiagen) was reverse transcribed with AMV reverse Transcriptase (Promega, Madison, WI, USA) according to the manufacturer’s instructions. cDNAs were amplified by qPCR reaction using GoTaq® qPCR Master Mix (Promega) and the reaction was carried out in BioRad-iQ-iCycler. The specific primer pairs are listed in Table 1. The results were analysed with CFX Manager software (Biorad) and the relative amounts, obtained with the 2^(−ΔCt)^ method, were normalized with respect to the housekeeping gene L34. Statistical significance was determined with a t-test with GraphPad Prism version 8.0 (La Jolla, CA, USA). Differences were considered significant at *P* < 0.05 (*p < 0.05; **p < 0.01; ***p < 0.001).

**Table 1:**
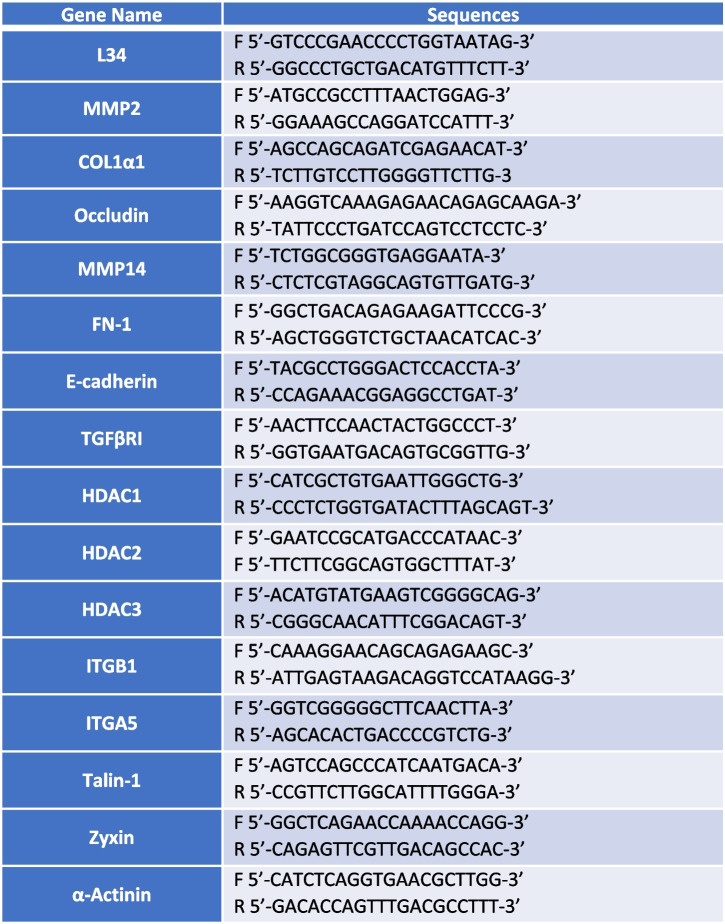
List of primers used in this study

### Biochemical HDAC1, − 3, − 4, − 6, − 8 isoform evaluation

MC3105 was tested against human recombinant HDAC1, − 3, − 4, − 6, and − 8, in 10-dose mode with 3-fold serial dilution starting from 200 μM solutions to determine their inhibitory potency (Table 2). The fluorogenic monoacetylated peptide from p53 residues 379–382 (Arg-His-Lys-Lys(Ac)AMC) was used to detect inhibitory activity against HDAC1–3, − 6, while for HDAC8 the diacetylated peptide from p53 residues 379–382 (Arg-His-Lys(Ac)-Lys(Ac)AMC) was used. For HDAC4 (class IIa HDACs), the fluorogenic class IIa (Boc- Lys(trifluoroacetyl)-AMC) substrate was employed [32, 33].

**Table 2:**
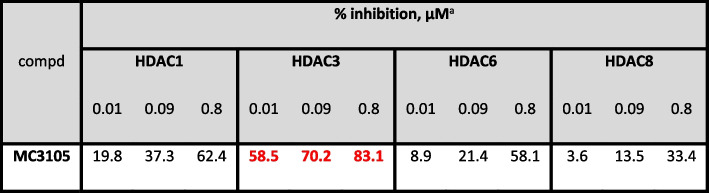
Biochemical HDAC1, − 3, − 4, − 6, − 8 isoform evaluation

^a^Against HDAC4, MC3105 displayed 4% of inhibition at 0.8 μ(M)

### siRNA-mediated knockdown and Talin-1 ectopic expression

siGENOME SMARTpool siRNAs HDAC1(3065), HDAC2 (3066) and siRNA control were purchased from Dharmacon. siRNA against human TALIN-1 5’CUCCAAGAGUAUUAUUAAU3’ and siRNA against human HDAC3 (Silencer® Select HDAC3 cat.4390825) were from Thermo Fisher.

200 × 10^3^ MeT5A cells were seeded on 6-well plates 24 hours before transfection. Cells were transfected with 100 pmol of siRNAs and 3.5 μl Lipofectamine® RNAiMAX Reagent (Thermo Fisher Scientific) were diluted in two different tubes with 400 μl Opti-MEM (Gibco-Life Technologies).

The two solutions were mixed gently and were incubated for 10–20 minutes at room temperature. Transfection solutions were added to cells with 1.2 ml of supplemented medium. To improve the efficiency, a second transfection was performed after 48 hours from the first transfection for 24 hours.

The efficiency of transfection was evaluated using RT-PCR or Western Blot.

Talin-1 was ectopically expressed using EYFP-Talin1-FERM (Plasmid #110565) from Addgene (Watertown, USA).

### Adhesion assay

MeT5A cells and primary MCs were plated at confluence on angiogenesis ibidi plates (Gräfelfing, Germany) or on 384-well ViewPlate optical bottom plates (for automated adhesion assays). MeT5A here stimulated with TGFβ1 (2 ng/ml) in combination with IL-1β (0.5 ng/ml) for 48 hours. MeT5A and primary MCs were treated with MS-275 for 72 hours. 5 × 10^3^ SKOV-3 GFP or OVCAR-3 GFP cells were added to the MC monolayer; after 30 min the wells were gently washed to eliminate non-attached cells. Remaining cells were then fixed in 4% paraformaldehyde. Samples were stained with 4′,6-diamidino-2-phenylindole (DAPI) and phalloidin.

### Mesothelial clearance assay

Spheroids were generated as in [34]. OVCAR-3 and SKOV3 cell spheroids were formed by incubating 1 × 10^3^ cells per well in a 96-well U-bottom-shaped culture dish with a cell-repelling surface (Cat# F202003, faCellitate) at 37 °C for 96 hours in the presence of MS-275 (1 𝜇M for OVCAR3, 2.5 𝜇M for SKOV3 cells). The MC monolayer was prepared by plating 30 × 10^3^ MeT5A cells per well 48-well microplate. Cells were treated with TGFβ1 and IL-1β for 48 hours and with MS-275 for 72 hours. Spheroids were transferred to the dish with the MC monolayer and the images of two cell populations were taken. Spheroid-induced mesothelial clearance was monitored by time-lapse microscopy using an epifluorescence inverted microscope Celldiscoverer 7 (Carl Zeiss AG, Oberkochen, Baden-Württemberg, Germany) equipped with a cage incubator for temperature and CO_2_ control. Fluorescence and phase-contrast images (5x objective) were collected for each experimental condition for 24 hours at 4 hours intervals. At 24 hours, the non-fluorescent area in the MC monolayer underneath the spheroid was measured by Celldiscoverer 7 (Carl Zeiss) and normalized to the initial spheroid area. Experiments were conducted at least in triplicate.

### ECM decellularization

For the detection of FN-1 from a cell-free matrix, MeT5A cells were grown past confluence in a 12-well plate with TGFβ1 (2 ng/ml) in combination with IL-1β (0.5 ng/ml) for 48 hours and MS-275 (250 nm) for 72 hours. Cell cultures were decellularized and the cellular material was removed as previously described [35]. Decellularized matrices and control (CTR) cells were prepared for the immunofluorescence as below, and all samples were stained with anti-FN antibody and DAPI. Confocal images were acquired using Celldiscoverer 7, Carl Zeiss AG.

### Confocal microscopy and immunofluorescence

Cells were fixed for 20 minutes in 4% formaldehyde in PBS, were permeabilized in 0.2% Triton X-100/PBS for 5 minutes and were blocked with 2% BSA for 20 minutes.

Angiogenesis Ibidi plates used for adhesion assay were mounted using ibidi Mounting Medium (ibidi). Confocal images were acquired using the Olympus iX83 FluoView1200 laser scanning confocal microscope.

For automated adhesion assays, cocultures were plated and treated as indicated above on 384-well ViewPlate optical bottom plates (PerkinElmer), processed and stained as described, and acquired on a spinning disk automated confocal microscope Opera HCS II (PerkinElmer). Images were analyzed for mesothelium layer confluency and relative tumor cell adhesion using the Columbus (TM) platform.

Immunostained coverslips were mounted in Prolong Gold antifade (Life Technologies) and examined with confocal microscopes (Leica TCS SP2, Wetzlar, Germany and Celldiscoverer 7, Carl Zeiss AG). Digital images were acquired with the Leica software and the image adjustments and merging were performed using the appropriate tools of ImageJ software.

For the estimation of FN-1 fiber secretion and deposition, quantitative image analyses from confocal immunofluorescence acquisitions were deployed using open-source Fiji/Image J 1.50e 64x. Fiberness measures the amount of fiber-like structures in an image, providing a readout that considers both the density of fibers and their length independently of orientation, and the macro has been described in [36]. Briefly, after noise reduction, structural information from the eigen values of the Hessian matrix is used to apply a Frangi vesselness filter to enhance very thin, almost unidimensional tubular structures. The output is a fiber-enhanced image in which each pixel contains a fiberness score. M0 readout was computed as the mean fiberness score in the whole image, and values were normalized to the average of the untreated samples. A second approach was to manually segment a perinuclear ring region with a thickness 0.4 that of the largest dimension of nuclei, and compute the % of total FN-1 staining intensity this represents.

JACoP plugin on ImageJ was used to quantify the colocalization of active β1-Integrin and total β1-Integrin with Mander’s coefficient (M1-M2). M1 is defined as the ratio of the “summed intensities of pixels from the green channel for which the intensity in the red channel is above zero” to the “total intensity in the green channel” and M2 is defined conversely for red. In this case we use M2.

### Immunohistochemistry

Patient serial tissue sections were deparaffinized and heated to expose the hidden antigens using Real Target Retrieval Solution containing citrate buffer, pH 6.0 (Sigma-Aldrich). Endogenous peroxidase was blocked with REAL Peroxidase-Blocking Solution (Dako). Samples were stained using primary antibodies to detect PDPN (Origine Technologies), FAP (Abcam), HDAC1 (Cell Signalling) and HDAC2 clone 3F3 (EMD Millipore). A biotinylated goat anti-rat IgG, anti-rabbit IgG or anti-mouse IgG (Vector Laboratories) was applied to detect primary antibodies. Complexes were visualized using the VECTASTAIN Elite ABC Reagent, Peroxidase, R.T.U. (Vector Laboratories) and 3,3′-diaminobenzidine (DAB; Dako) as chromogen. Finally, tissue sections were counterstained with hematoxylin.

Mouse paraffin sections (3 μm thick) were stained with haematoxylin and eosin (H&E) for histological evaluation. For immunohistochemical analysis, deparaffinized tissues were heated to expose the hidden antigens using Real Target Retrieval Solution containing citrate buffer, pH 6.0 (Sigma-Aldrich). Endogenous peroxidase was blocked with Real Peroxidase-Blocking Solution (Dako). The Mouse-Over-Mouse Polymer IHC Kit (Abcam) was applied according to the manufacturer’s instructions to detect the following primary mouse monoclonal antibodies: anti-α-SMA (Sigma-Aldrich, St Louis, MO) and anti-pan-cytokeratin (clone PCK-26; Sigma-Aldrich). To detect FN-1, tissue sections were incubated with a primary antibody (Abcam) and a secondary biotinylated goat anti-rabbit IgG (Vector Laboratories). The complex was visualized using the VECTASTAIN Elite ABC Reagent, Peroxidase, R.T.U. (Vector Laboratories). Finally, tissue sections were revealed using DAB as chromogen and finally counterstained with haematoxylin. Images were captured with a digital camera coupled to a brightfield microscope and 2–4 arbitrary fields (magnification × 200) per sample were considered for quantification.

### Flow cytometry analysis of cell surface β1-integrin expression and activation

MeT5A cells were cultured in 6-well culture plates for 72 hours with or without MS-275 (250 nM) before flow cytometry analysis. Cells were then detached with Trypsin/EDTA solution, washed twice in FBS-free RPMI and incubated either with anti-β1 integrin mAb TS2/16 (which detects all β1 integrin molecules, regardless of their activation state) or with mAb HUTS21 (which detects only activated/high affinity β1 integrin molecules). For mAb TS2/16, cells were incubated for 30 min at 4 °C in FBS-free RPMI-1640, and for mAb HUTS21, cells were incubated for 15 min at 37 °C in an incubation buffer with defined cation conditions (20 mM HEPES, 150 mM NaCl, 2 mg/mL glucose, 1 mM MgCl_2_ and 0.5 mM CaCl_2_). After incubation with primary antibodies, cells were washed three times in RPMI-1640 and incubated with the secondary polyclonal antibody DyLight™ 649-conjugated Goat anti-mouse IgG (Thermo Fisher) for 30 min at 4 °C. Cells were washed three times in FBS-free RPMI-1640 and then fixed in 2% formaldehyde solution in PBS. Fluorescence was measured using a FACScan™ flow cytometer (Beckton-Dickinson, NJ, USA). Cytometry data was processed using the FlowJo (v10 version) software.

### Proteomics

Mesenchymal-like Met5A (*n* = 2) or primary MCs cells (*n* = 3), either treated with MS-275 (250 nM for 72 hours) or untreated, were lysed in RIPA Buffer. The proteomic analysis was conducted as in [37].

### Mouse model of EOC peritoneal metastasis

A total of 15 Swiss *nu/nu* 9/10-week-old female mice were used in this study (Charles River Laboratories, Barcelona, Spain). The experimental protocol was in accordance with the National Institutes of Health Guide for Care and Use of Laboratory Animals and was approved by the Animal Ethics Committee of the Unidad de Experimentación Animal del Centro de Biología Molecular Severo Ochoa – CSIC (Madrid, Spain; ethic approval number: 863/2019), as well as by Community of Madrid (Madrid, Spain; PROEX number 273/19). Mice were housed in cages provided with food and water ad libitum.

A total of 5 × 10^6^ SKOV-3-luc-D3 cells expressing luciferase (SKOV3-luc-D3 Bioware; Caliper Life Sciences, Hopkinton, MA, USA) were inoculated into the peritoneal cavity of mice. The human ovarian carcinoma cell line SKOV-3-luc-D3 was grown in McCoy’s 5A medium (Sigma-Aldrich) supplemented with 10% FBS and 0.8 mg/mL of geneticin (G418; Gibco) as a selection agent. Mice were randomly grouped to receive intraperitoneal pretreatment of a total of 500 μL of 2% DMSO combined with 30% polyethylene glycol (PEG)-300 (Sigma-Aldrich) in phosphate buffered saline (PBS) (vehicle group; *n* = 8) or 15 mg/Kg MS-275 2% DMSO 30% PEG-300 in PBS (MS-275 group; *n* = 7), 2 days before the SKOV-3-luc-D3 inoculation. After intraperitoneal injection of SKOV-3-luc-D3 cells, mice received treatment 3 times a week for 5 weeks. Upon cervical injection of 200 μL of D-luciferin (Perkin-Elmer, Waltham, MA, USA) and anesthesia with inhaled isoflurane (Isoflutek 1000 mg/g), tumor-produced bioluminescence signal was monitored with IVIS Lumina II (Perkin-Elmer) twice weekly for 40 days. At the end of the experiment, mice were sacrificed upon CO_2_ inhalation and the peritoneal luciferase signal was measured. Bioluminescence images were quantified using Living Image 4.7.3. Software (Caliper LS).

### Statistical analysis

Statistical significance was determined with a *t*-test using GraphPad Prism version 5.0 (La Jolla, CA, USA). Differences were considered significant at *P* < 0.05.

Perseus software (version 1.6.7.0) after log2 transformation of the intensity data was applied to proteomic study. Statistical analysis was conducted on proteins identified in 100% of the samples. To improve visualization, a z-score plot and a cluster heat map were generated. The correlation analysis between Met5A and primary MCs proteomes in CTR and MS-275 treated samples was performed by calculating Pearson’s correlation coefficient. Results were considered statistically significant at *P* < 0.05 Gene ontology enrichment analysis of biological processes, molecular functions and cellular components were performed by PANTHER software using Fisher’s exact test and applying the false discovery rate calculation as a correction for multiple testing.

## Results

### Treatment with MS-275 promotes MMT reversal and limits EOC cells/MCs adhesion

**Fig. 1:**
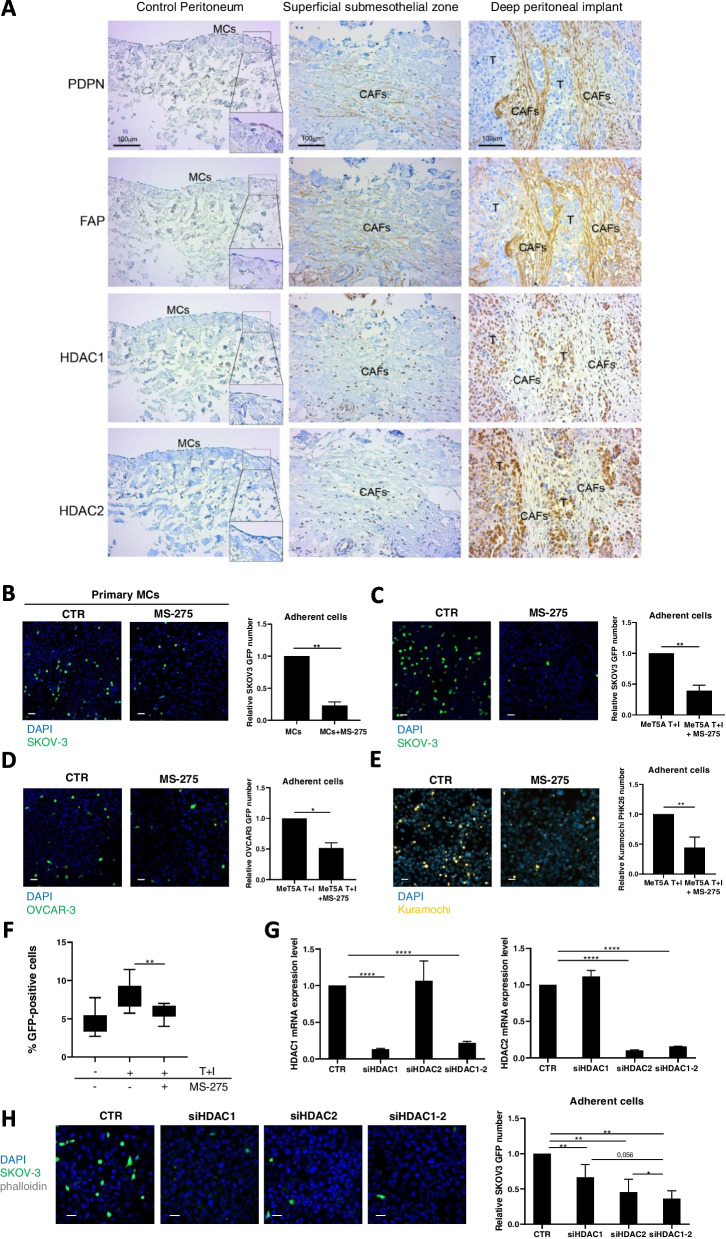
EOC peritoneal metastasis biopsies show HDAC1 increased expression in MC-derived CAFs and HDAC1–2 inhibition limits mesenchymal-like MCs/EOC adhesive interactions. **A** left, serial sections of a control peritoneum show a conserved MC monolayer negative for HDAC1 and HDAC2. Insets show a higher magnification of the delimited areas. **A** Middle, serial sections of a sub-mesothelial compact zone in an EOC patient with peritoneal metastasis show areas of Podoplanin (PDPN), Fibroblast Activation Protein (FAP) and nuclear HDAC1 and HDAC2 co-localization. Right, Representative images of PDPN and FAP staining of spindle-like cells surrounding deep tumor nodules. Nuclear HDAC1 and HDAC2 staining overlap with areas of accumulation of MC-derived CAFs. Tumor cells are also HDAC1 and HDAC2 positive. Scale bar: 100 μm; CAFs: carcinoma-associated fibroblasts; T: tumor. **B** Representative images of GFP-labelled SKOV3 cells adhering to primary MC monolayers; **C** GFP-SKOV3 cells adhering to MeT5A cell monolayers; **D** GFP-OVCAR-3 cells adhering to MeT5A cells monolayers; **E** PHK26-stained Kuramochi cells adhering to MeT5A cells monolayers. Nuclei are stained with DAPI (blue). CTR: control treatment. MeT5A cells monolayers were at 100% confluence at the time of the experiment. Scale bar: 25 μm. Quantifications are shown at the right of each figure. MeT5A cells were pretreated with TGFβ1 in combination with IL-1β (T + I), and treated or not with MS-275 (250 nM) for 72 hours. Results are shown as relative number of adherent SKOV3 cells. 3 fields for each sample were analyzed. **F** Images of GFP-SKOV3 cells adhesion to MeT5A cells were acquired with a spinning disk automated confocal microscope and analyzed using Columbus (TM) platform considering relative tumor cells number. Results are shown as percentage of attached GFP-SKOV3 cells out of total seeded cells. **G** qRT-PCR showing genetic silencing of HDAC1, HDAC2 alone and HDAC1 in combination with HDAC2 from total RNA of MeT5A cells used for the experiment shown in **H**. Bars represent means±SEM of 3 experiments. **H** Adhesion assay showing adhesion of GFP-labelled SKOV3 cells to siHDAC1, siHDAC2 and siHDAC1-HDAC2 MeT5A cells. Representative images are shown on the left. Scale bar: 10 μm. Quantification of the experiment is shown on the right. Each experiment was performed at least 3 times in triplicate. Differences were considered significant at *P* < 0.05 (*p < 0.05; **p < 0.01; ***p < 0.001)

The central hypothesis of this study is that HDAC1–2 activity promotes EOC cell adhesiveness to MCs as the first step of EOC peritoneal metastasis; this hypothesis is based on the observation of HDAC1–2 overexpression in both mesothelium derived-CAFs (podoplanin, PDPN and fibroblast activation protein, FAP double-positive cells) and EOC from EOC patients (Fig. 1A) and on the ability of HDAC1 inhibition to revert MMT features in mesenchymal-like MCs [24].

Adhesion of different GFP-labeled platinum-resistant EOC cells (SKOV3 and OVCAR-3) and platinum-sensitive EOC (Kuramochi cells) to the MC line MeT5A or primary MCs was analyzed in static adhesion assays (Fig. 1B-E). As previously reported [10], the acquisition in MCs of mesenchymal-like features by treatment with TGFβ1 in combination with IL-1β facilitates the adhesion of EOC cells (Suppl. Fig. S1A). In all the experimental conditions examined, treatment of MCs with MS-275 (250 nM) significantly inhibited EOC adhesion to MCs. The efficiency of MS-275 treatment on MCs was also confirmed with automated adhesion assays (Fig. 1F).

Interestingly, the inhibition of EOC/MC adhesion was also found when EOC lines were pretreated with MS-275, and was more evident when both cell types were pretreated with this HDAC1–3 inhibitor **(**Suppl. Fig. 1B-C**)**.

**Fig. 2:**
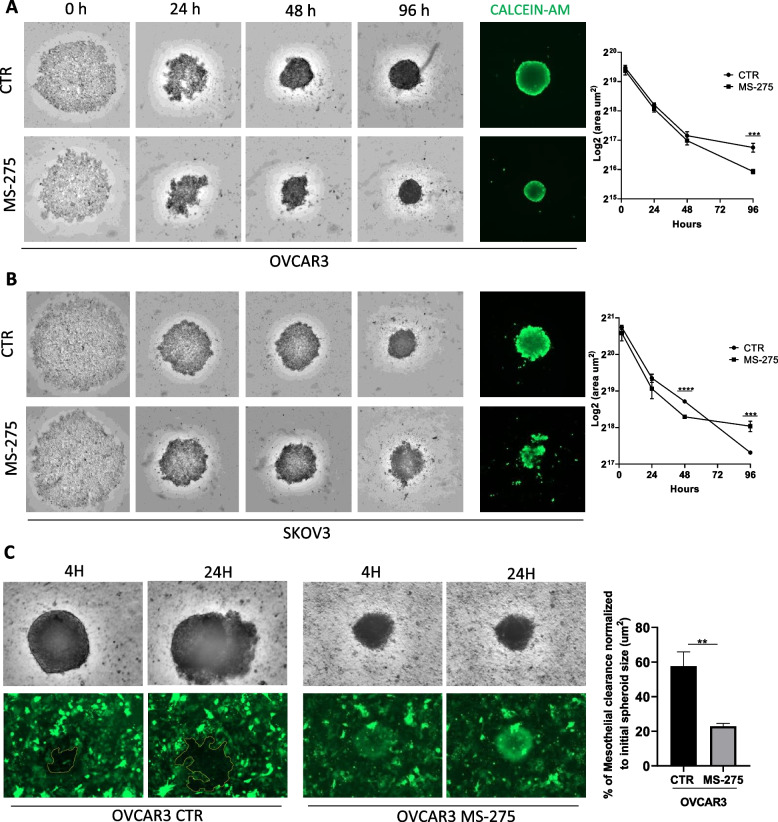
Treatment with MS-275 impacts on EOC 3D spheroid generation and on spheroid mediated peritoneal clearance (**A**) OVCAR-3 cells 3D spheroids were generated in the presence of MS-275 (1 μM). **B** SKOV3 cells 3D spheroids were generated in the presence of MS-275 (2.5 μM). Images were taken at 0, 24, 48 and 96 hours. Quantification of the area is shown at the right of the images. Cell vitality at 96 hours was analyzed by staining with Calcein AM. Representative images are shown from one experiment of 4 performed. **C** Images show mesothelial clearance induced by OVCAR-3 spheroids treated or not with MS-275 (1 μM) after 4 and 24 hours. The chart at the right of the images represents the ratio in percentage between the area of ​​the gap formed by the spheroid on the mesothelial monolayer (time 24 hours, labeled in yellow) and the spheroid area (time 0 hours). Representative images are shown from one experiment of 6 performed. Differences were considered significant at *P* < 0.05 (*p < 0.05; **p < 0.01; ***p < 0.001)

The results obtained using MS-275 were confirmed by specific genetic silencing. As shown in Fig. 1G-H, both HDAC1 and HDAC1–2 genetic silencing significantly reduced EOC cells/MCs adhesion. Interestingly, genetic silencing data suggest a cooperation between HDAC1 and HDAC2 in controlling MCs/EOC adhesion (Fig. 1H**)**. The involvement of HDAC3 in this EOC cells/MCs adhesion was ruled out using MC-3105, a specific HDAC3 pharmacological inhibitor (Suppl. Fig. 2A). This compound was demonstrated to be a highly selective single-digit nanomolar HDAC3 inhibitor with selectivity index ranging from 120-fold to 27,000-fold over HDAC1 and HDAC4 isoforms, respectively (Table 2). In addition, HDAC3 genetic silencing did not significantly modulate EOC/MC adhesion (Suppl. Fig. 2B). Moreover, mesothelial clearance experiments were performed. Tumor spheroids used in this assay are multicellular aggregates found in vivo and able to dynamically adhere to the mesothelial membrane favoring the metastatic process [38, 39]. Interestingly, MS-275 treatment interfered with the formation and growth of spheroids between 48 and 96 hours of treatment. Intracellular acquisition of intravital dye (CALCEIN AM) showed the vitality of both OVCAR-3 and SKOV3 cells at 96 hours of treatment (Fig. 2A-B). As expected, mesothelial clearance was significantly inhibited by MS-275 treatment (Fig. 2C).

Overall, these results demonstrate that HDAC1–2 inhibition was sufficient to limit EOC cells/MCs adhesion in both static and dynamic assays.

### Proteomic analysis of mesenchymal-like MeT5A cells and primary MCs treated with MS-275 reveals a profound impact on the expression of adhesion molecules, cytoskeleton regulators and extracellular matrix proteins

In order to elucidate the molecular mechanisms underlying the observed effect, the proteome from mesenchymal-like MeT5A cells left untreated or treated with MS-275 was analyzed by quantitative mass spectrometry analysis.

**Fig. 3:**
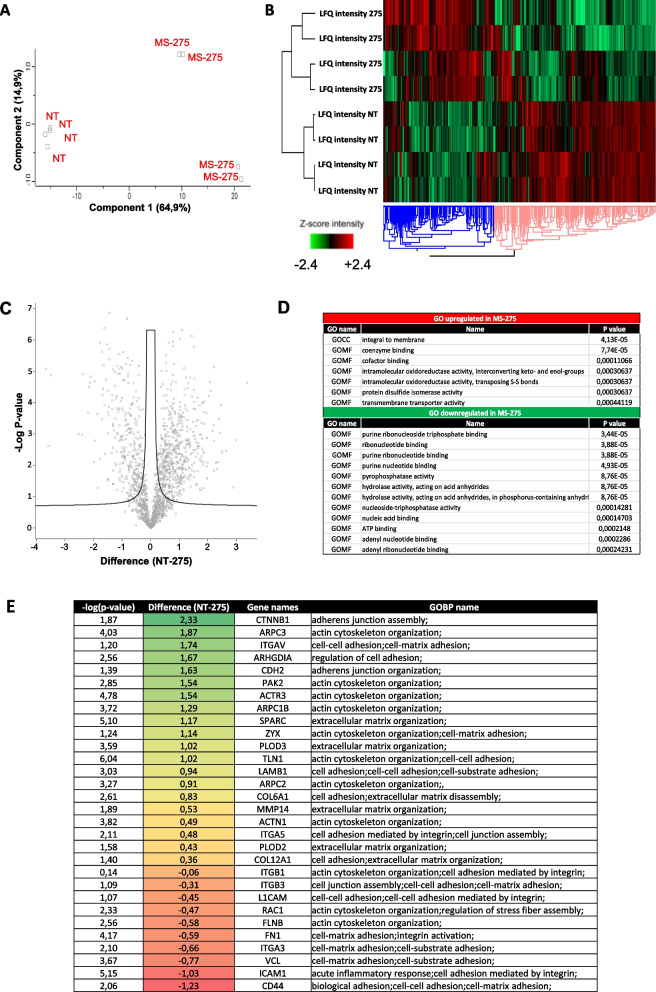
Treatment with MS-275 modifies the proteome of mesenchymal-like MeT5A cells. Mesenchymal-like MeT5A cells were left untreated or treated for 72 hours with MS-275 (250 nM) (*N* = 2). Cells were lysed with RIPA buffer and quantified by Bradford assay. Total lysates were digested and separated in 8 fractions based on proteins’ hydrophobic properties. Separated fractions were analysed by label-free liquid chromatography-mass spectrometry (nLC-MS/MS). **A** Principal component analysis (PCA) of the LFQ intensities obtained in NT and MS-275 treated sample datasets. **B** Heat map of differentially expressed proteins in NT and MS-275 samples. LFQ intensities were expressed in z-score values (range of intensity z-score: ±2.4). Up-regulated and down-regulated proteins are expressed in red and green scales respectively. Hierarchical clustering was performed using Euclidean distance and average linkage using the Perseus software. **C** Volcano plots comparing NT (left panel) and MS-275 (right panel) upregulated proteins. Black curves represent the significance threshold at false discovery rate (FDR) of 0.05 and S0 of 0.1. **D** Gene Ontology enrichment analysis performed by Perseus software on differentially expressed proteins between NT and MS-275 datasets. GOCC: Gene ontology cellular components; GOMF: Gene ontology molecular functions (**E**) Table showing selected identified proteins belonging to specific Gene ontology biological processes (GOBP) shown in the right column

Principal component analysis (PCA) indicated that untreated and MS-275-treated mesenchymal-like MeT5A are distributed in distinct groups (Fig. 3A). Hierarchical clustering classified the samples into two groups based on differentially expressed proteins, as represented by Heat map visualization (Fig. 3B). Volcano plot representing differential expression analysis (DEA) identified 859 proteins with FDR < 0.05 (553 were upregulated by MS-275 treatment, 306 were downregulated) (Fig. 3C). Specifically, gene ontology enrichment analysis revealed integral membrane proteins as the functional class of proteins most significantly upregulated (Fig. 3D). The analysis of specific modulated proteins identified many adhesion molecules and ECM proteins as upregulated in MeT5A treated group. Among downregulated proteins, Actin cytoskeleton organization related proteins as well as ECM regulation proteins were found (Fig. 3E). Besides MeT5A cells, a proteomic analysis was also performed on primary MCs (Suppl. Fig. 3A-E). Volcano plot representing differential expression analysis (DEA) identified 269 proteins with FDR < 0.05 (88 were upregulated by MS-275 treatment, 181 were downregulated). Similarly to MeT5A cells, Actin cytoskeleton organization related proteins as well as ECM regulation proteins were found significantly modulated. A comparison between MeT5A cell and primary MC proteomes revealed a significant positive Pearson correlation between proteins expressed both in CTR (r:0.63) and MS-275 treated samples (r:0.68) (Suppl. Fig. 4A-B). Venn diagrams show 71 proteins significantly downregulated by treatment with MS-275 common to MeT5A and primary MCs, whereas 14 proteins were upregulated (Suppl. Fig. 4C-D). The complete list of proteins significantly modulated common to MeT5A and primary MCs is shown in Suppl. Fig. 5, 6. Interestingly, Actin cytoskeleton organization related proteins were similarly downregulated in the two subsets.

The results obtained by proteomic analysis were the starting point for the further identification of molecular mechanisms underlying HDAC1/2-mediated EOC cells/MCs adhesion.

### MS-275 perturbs α5β1 integrin activity in MCs

**Fig. 4:**
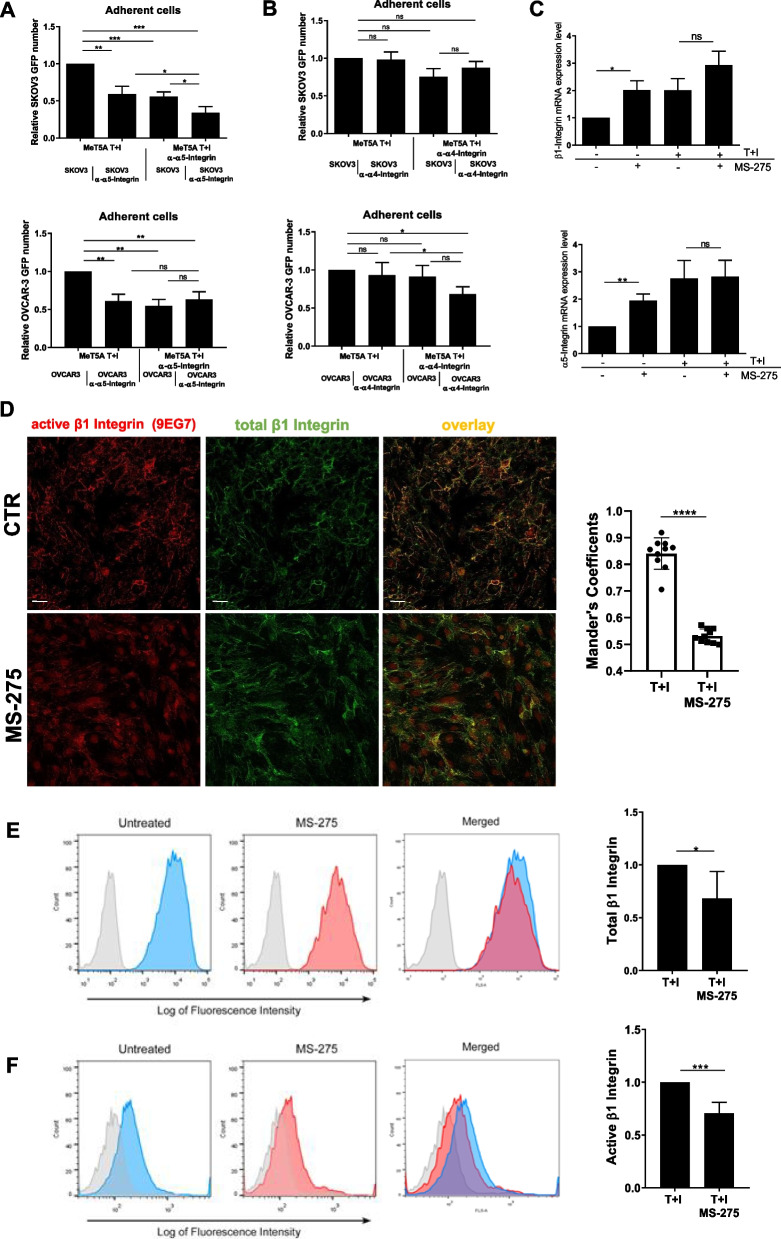
Effects of MS-275 on β1 Integrin expression and activity (**A-B**) Adhesion assays of mesenchymal-like MeT5A pretreated with anti-Integrin α5 and -Integrin α4 blocking antibodies. Results are shown as relative number of adhered EOCs (GFP-SKOV3 cells top, GFP-OVCAR-3 cells, bottom) on the MeT5A monolayer. Adherent SKOV3 cells were analyzed in 3 fields/sample. Each experiment was performed at least 3 times in triplicate. Mesenchymal-like MeT5A cells treated with MS-275 (250 nM) for 72 hours. **C** RT-qPCR showing the expression of β1 and α5 Integrin subunits from total RNA of mesenchymal-like MeT5A cells treated with MS-275 (250 nM) for 72 h. Bars represent means±SEM of 5 independent experiments. **D** Immunofluorescence showing mesenchymal-like MeT5A cells treated with MS-275 (250 nM) for 72 hours. Fixed cells were stained with an antibody against total β1 Integrins or against active β1 Integrins (9EG7). The quantification of the experiment is shown on the right. Mander’s colocalization M2 coefficients were measured using the JACoP plugin on ImageJ. At least 10 images were quantified per experiment. Confocal images are shown from one representative experiment of three performed. Scale bar: 20 μm, **E**,** F** left, flow cytometry experiments showing the plasma membrane expression total β1 Integrin (**E**) and of active β1-Integrin detected using the monoclonal antibody HUTS21 (**F**). The fluorescence intensity profiles measured through flow cytometry depict a representative experiment. Active β1-Integrin in untreated MeT5A cells appears in blue, whereas in MS-275 treated cells (250 nM) it appears in red. Light-grey profiles depict negative controls. Right, histograms show mean fluorescence intensities (MFI) of β1 Integrin (**E**) and active β1 Integrin stainings (**F**). Bars represent means ± SEM of 5 experiments. Differences were considered significant at *P* < 0.05 (*p < 0.05; **p < 0.01; ***p < 0.001; **** *p* < 0.0001)

To analyze molecular mechanisms underlying the observed inhibition of EOC cells/MCs adhesion upon MS-275 treatment, we focused on FN-1 receptors α4β1 and α5β1 integrins, previously demonstrated as the main adhesion receptors implicated in EOC/MCs adhesion in murine and human experimental models [10, 40]. Using specific inhibitory antibodies, α5β1 Integrin was demonstrated to play a main role in MeT5A/SKOV3 cell adhesion, whereas the effect of the anti-α4 blocking antibody was not significant (Fig. 4A-B, top). Interestingly, when analyzing the effect of these antibodies on Met5A/OVCAR3 adhesion, combined inhibition scored significative also for α4β1 Integrin, (Fig. 4A-B, bottom). The effect of α5β1 Integrin inhibition in MC highlights an active role of mesothelium Integrins in the regulation of MC/EOC cell adhesion (Fig. 4A). The specific effect of MS-275 on α5β1 Integrin expression was then analyzed. Unexpectedly, a significative induction of both α5 and β1 mRNAs was observed in MeT5A cells upon MS-275 treatment (Fig. 4C top-bottom), thus ruling out a transcriptional repression. We then focused on β1 Integrin activity. To this purpose, two different antibodies (9EG7 and HUTS21) specific for the activation state of β1 Integrins were used [29, 41]. In both cases, immunofluorescence staining and flow cytometry analyses showed a decrease of β1 activation after treatment with MS-275 both in MeT5A and in primary MCs (Fig. 4D-E and Suppl. Fig. 7). Therefore, treatment with MS-275 hampers β1 Integrin activity in mesenchymal-like MCs.

### Aberrant expression and organization of FN-1 upon MS-275 treatment in MCs

**Fig. 5:**
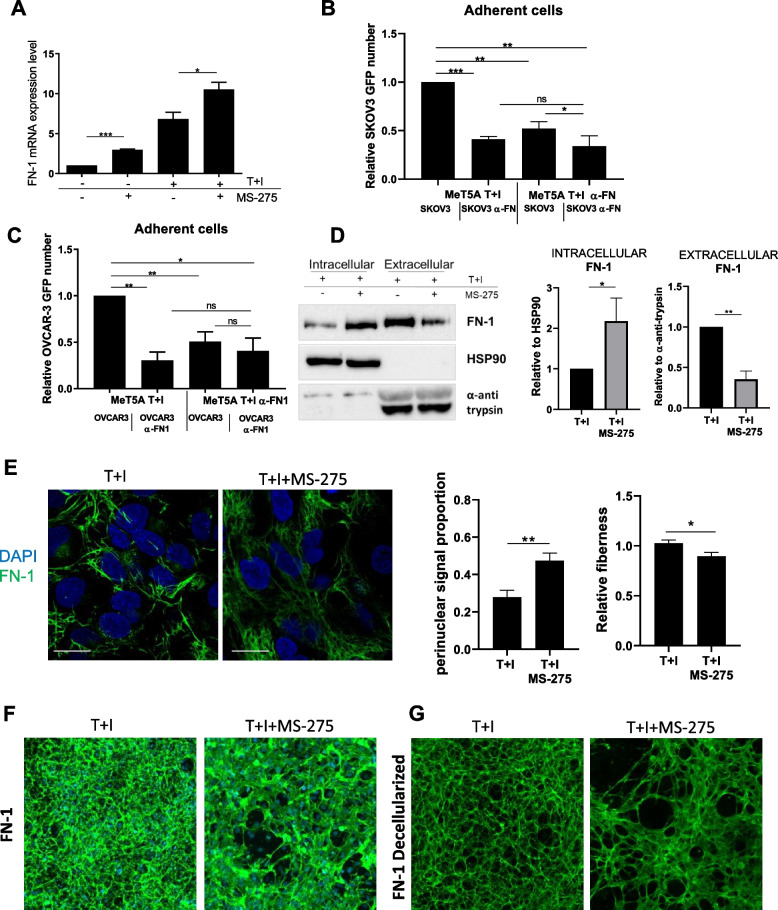
Effects of MS-275 on FN-1 expression and extracellular secretion (**A**) RT-qPCR experiment showing FN-1 expression from total RNA of mesenchymal-like MeT5A cells treated with MS-275 (250 nM) for 72 hours. Bars represent means±SEM of 5 experiments (**B-C**) Adhesion assay of GFP-SKOV3/MeT5A cells (**B**) and GFP-OVCAR-3/MeT5A cells (**C**) treated with an anti-FN-1 blocking antibody. Results are shown as relative number of adherent SKOV3 cells. 3 fields for each sample were analyzed. This experiment was performed 3 times. **D** Representative Western blot showing expression FN-1 from cell lysates of mesenchymal-like MeT5A cells treated as above or from cell supernatant. HSP90 and anti-anti trypsin were used as a loading control. One experiment is shown of 3 performed. Quantifications are shown in the right. **E** Immunofluorescence of mesenchymal-like MeT5A cells treated with MS-275. Cells were fixed, permeabilized and stained with an anti-FN-1 antibody. Nuclei are stained with DAPI (blue). Confocal images are shown from one representative experiment of four performed. Scale bar: 10 μm, Quantifications of FN-1 perinuclear proportion and fiberness are shown on the right of the figure. Differences were considered significant at *P* < 0.05 (*p < 0.05; **p < 0.01; ***p < 0.001; **** *p* < 0.0001). **F**,** G** FN-1 staining of MeT5A cells fixed and permeabilized (**F**) or decellularized matrices (**G**) after treatment with MS-275 (250 nM) for 72 h. Nuclei are stained with DAPI. Decellularized matrices are shown on the right. Representative images are shown from one of three experiments performed

FN-1 has been demonstrated to promote adhesion, invasion, proliferation, and metastasis of EOC cells [40]. Proteomic analysis from both MeT5A cells and MCs revealed an upregulation of FN-1 expression after treatment of MCs with MS-275, which was confirmed by RT-PCR and WB experiments (Fig. 5A), also after HDAC1–2 genetic silencing (Suppl. Fig. 8A and E). A blocking antibody to FN-1 significantly inhibited the adhesion of SKOV3 and OVCAR-3 to MeT5A cells, thus confirming the involvement of FN-1 in this experimental system of cell-cell adhesion (Fig. 5B-C). The observation that treatment with MS-275 inhibited EOC/MCs adhesion despite increased FN-1 expression prompted us to investigate further.

FN-1 secretion was analyzed by comparing FN-1 intracellular expression with FN-1 secreted in the culture supernatants. Treatment with MS-275 significantly increased the intracellular versus the secreted portion of FN-1(Fig. 5D).

Confocal microscopy experiments showed increased FN-1 perinuclear proportion and decreased fiber density/length upon treatment with MS-275, suggesting abnormal FN-1 secretion and remodeling (Fig. 5E). This result was confirmed by an assay of matrix decellularization, where reduced FN-1 deposition and altered distribution upon MS-275 treatment was observed (Fig. 5F-G).

These observations strongly suggest a role of HDAC1/2 inhibition in impairing MCs/EOC cell adhesion through an alteration of FN-1 secretion and extracellular remodeling.

### MS-275 perturbs α5β1 integrin activity by downregulating actin cytoskeleton regulators Talin-1, Zyxin and α-Actinin-1 in MCs

**Fig. 6:**
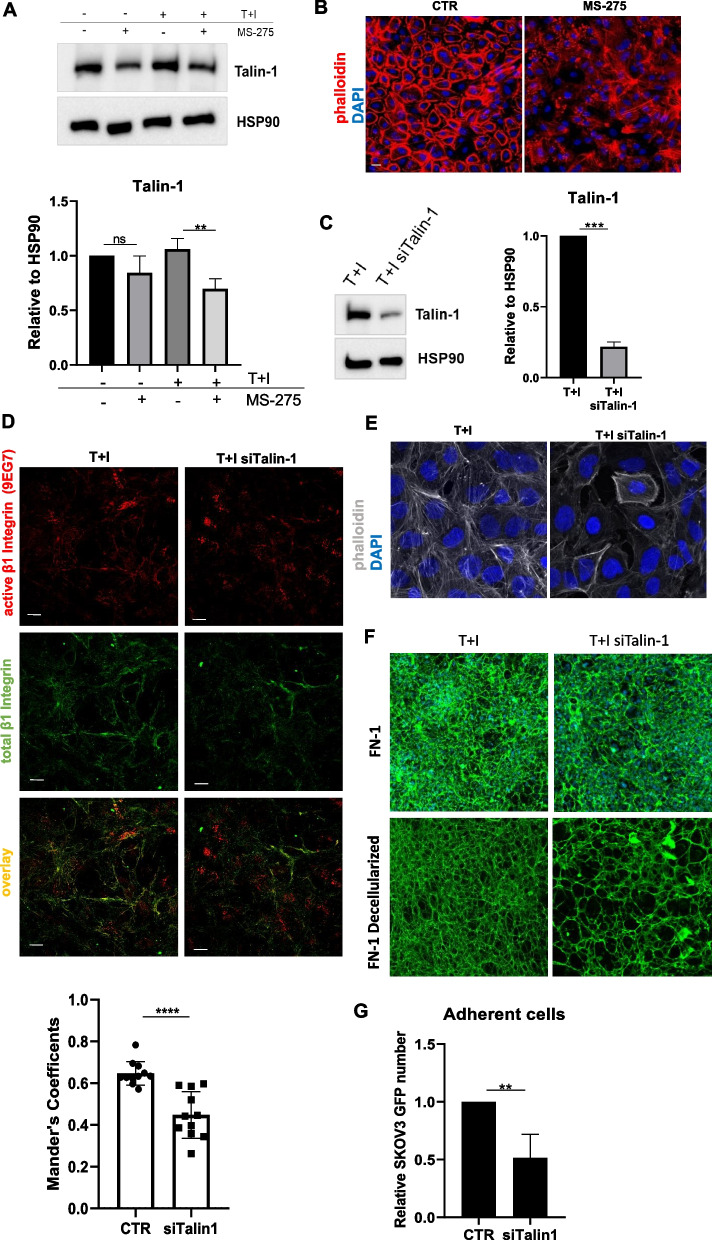
MS-275 hampers actin cytoskeletal organization and impacts the MeT5A/SKOV3 cell adhesion by downregulating Talin-1 expression. **A** Representative Western blot experiment showing expression of Talin-1 from cell lysates of MeT5A cells treated MS-275 (250 nM) for 72 hours. HSP90 was used as a loading control. One of three experiments is shown. Quantification of the experiments is shown below. **B** Immunofluorescence of primary mesenchymal-like MCs treated with MS-275 (250 nM) for 72 hours showing the expression of Actin filaments stained with phalloidin (red). Nuclei are shown in blue (DAPI). Scale bar: 10 μm. **C** Western blot showing Talin-1 expression in Talin-1 silenced MeT5A cells. HSP90 was used as a loading control. One of three experiments is shown. Quantification of the experiments is shown on the right. **D** Immunofluorescence showing mesenchymal-like MeT5A cells stained with an antibody against active β1 Integrins (9EG7) (top) or against β1 Integrins (bottom). Mander’s colocalization M2 coefficients were measured using the JACoP plugin on ImageJ. At least 10 images were quantified per experiment. The quantification of the experiment is shown at the bottom. Confocal images are shown from one representative experiment of three performed. Scale bar: 20 μm (**E**) Immunofluorescence of Talin-1 silenced MeT5A cells stained with phalloidin (grey) and DAPI (blue). Scale bar: 10 μm. Representative images are shown from one of three experiments performed. **F** FN-1 staining of decellularized matrices of MeT5A cells treated with genetically silenced for Talin-1 for 72 hours. Nuclei are stained with DAPI. Decellularized matrices are shown on the right. Representative images are shown from one of three experiments performed. **G** Adhesion assays on Talin-1 silenced MeT5A cells. Results are shown as relative number of adherent GFP-SKOV3 cells on Talin-1 silenced MeT5A monolayers. Adherent SKOV3 cells were evaluated in 3 fields/sample. Bars represent the means±SEM of four experiments. Differences were considered significant at P < 0.05 (*p < 0.05; **p < 0.01; ***p < 0.001; **** *p* < 0.0001)

**Fig. 7:**
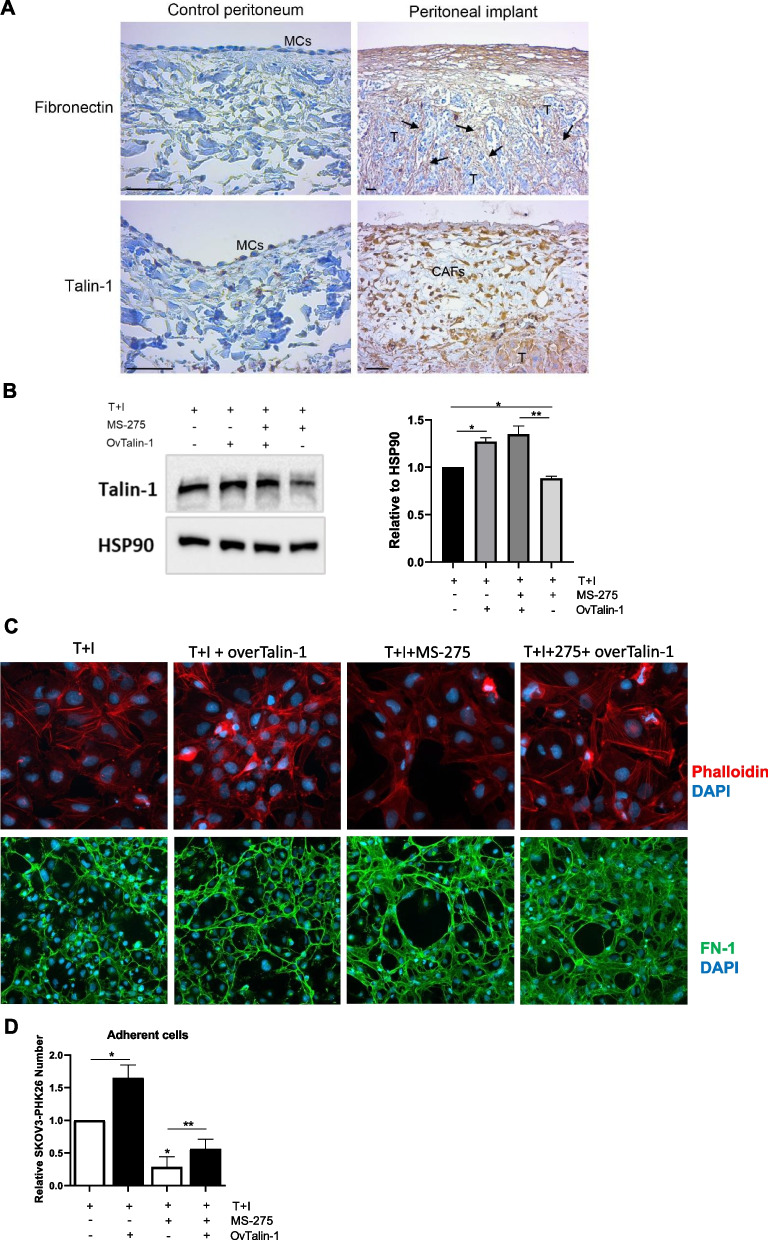
Talin-1 ectopic expression rescues the altered Actin polymerization, FN-1 extracellular distribution and MC/EOC adhesion upon treatment with MS-275 (**A**) A human control peritoneum shows a conserved MC monolayer negative for FN-1 (upper left). FN-1 (arrows) strongly stains the tumor surrounding stroma in a sample from a peritoneal carcinomatosis patient (upper right). The MC monolayer of a control peritoneal sample shows low levels of Talin-1 expression (bottom left). CAFs and tumor nodules accumulated in the sub-mesothelial compact zone of a patient sample show high staining for Talin-1 (bottom right). Scale bars: 50 𝜇m. MCs: Mesothelial cells; CAFs: carcinoma-associated fibroblasts; T: tumor. **B** Western blot showing ectopic expression of Talin-1 in Met5A cells treated with MS-275 (250 nM) for 72 hours. HSP90 was used as a loading control. One of three experiments is shown. Quantification of the experiment is shown on the right. **C** Immunofluorescence of Met5A cells treated with MS-275 (250 nM) for 72 hours where Talin-1 was ectopically expressed. Phalloidin staining to mark Actin (red) is shown in the top, FN-1 staining (green) is shown in the bottom. Nuclei were stained with DAPI. Representative images are shown from one of three experiments performed. **D** Adhesion assays on MeT5A cells where Talin-1 was ectopically expressed. SKOV3 cells were stained with PHK26. Results are shown as the relative number of adherent SKOV3 cells on Talin-1 ectopically expressed MeT5A monolayers. Adherent SKOV3 cells were evaluated in 3 fields/sample. Bars represent means±SEM of 3 experiments. Differences were considered significant at *P* < 0.05 (*p < 0.05; **p < 0.01; ***p < 0.001; **** *p* < 0.0001)

Since proteomic analysis demonstrated that many Actin-related molecules were downregulated upon treatment with MS-275 (Fig. 3E and Suppl. Fig. 3), we hypothesized that MS-275 could impair actin cytoskeleton rearrangements causal for α5β1 integrin activation and FN-1 secretion. First, we confirmed by RT-PCR and WB the downregulation of Talin-1, Zyxin and α-Actinin-1 in MCs upon exposure to MS-275, (Fig. 6A and Suppl. Fig. 9A-C) as well as after HDAC1–2 genetic silencing (Suppl Fig. 8 B-D and F). Accordingly, phalloidin staining showed a dramatic alteration of Actin polymerization upon treatment with MS-275 in primary MCs (Fig. 6B). In order to provide mechanistic evidence of the role of Actin cytoskeleton regulators in this experimental system, Talin-1 expression was genetically silenced (Fig. 6C). Immunofluorescence of Talin-1-silenced MCs showed alterations of Actin polymerization patterns (Fig. 6D), decreased β1integrin activation (Fig. 6E), a pattern of FN-1 extracellular deposition similar to that observed upon MS-275 inhibition (Fig. 6F) and significantly inhibited MeT5A/SKOV3 cell adhesion (Fig. 6G). As support for the biological relevance of our discoveries, we found increased expression of Talin1 and FN-1 in CAFs (bona fide of mesothelial origin) surrounding an EOC peritoneal implants (Fig. 7A). Accordingly, Talin-1 ectopic expression significantly rescued the altered actin polymerization (Fig. 7B), FN-1 secretion (Fig. 7C) and MeT5A/SKOV3 cell adhesion (Fig. 7D) observed upon treatment with MS-275. These results demonstrated that HDAC1/2 inhibition by treatment with MS-275 causes complex actin cytoskeleton alterations involving the downregulation of actin regulators including Talin-1, leading both to inhibition β1 activity and alterations of FN-1 secretion, eventually impairing MCs/EOC cells adhesion.

### Treatment with MS-275 hampers peritoneal metastasis in vivo

**Fig. 8:**
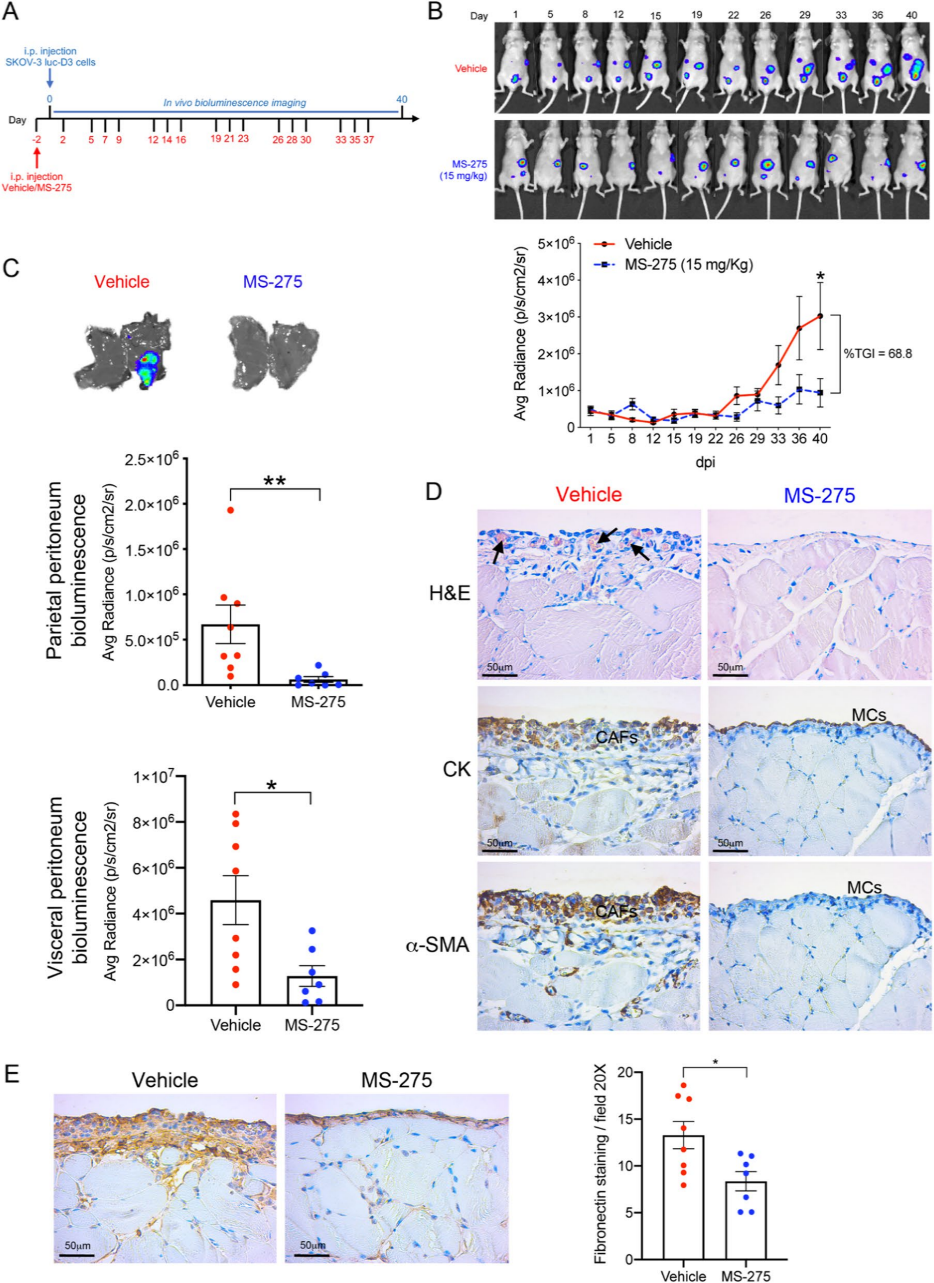
Evaluation of MS-275 treatment in a mouse model of EOC peritoneal metastasis. **A** In vivo experiment design. **B** Representative images of in vivo monitoring of SKOV3-luc-D3 cells in vehicle (*n* = 8) and MS-275 (*n* = 7) treated groups. Quantification of bioluminescence showed a significant tumor growth inhibition (TGI) in mice receiving MS-275 compared to the control group. **C** Representative images of parietal peritoneal tissues showing decreased tumor-emitting bioluminescence in MS-275 treated mice as compared to the vehicle group. Quantification of bioluminescence in parietal and visceral peritoneal tissues. The graph represents the mean average radiance (expressed as photons/s/cm^2^/sr) of SKOV3-luc-D3 cells ± SEM (*p < 0.05). **D** Parietal peritoneum samples were analyzed 5 weeks after i.p. injection of SKOV-luc-D3 cells. Haematoxylin & Eosin (H&E) staining shows a sub-mesothelial compact zone with accumulation of capillaries (arrows) in a vehicle mouse. A mainly conserved histological structure, without evidence of fibrosis and with a preserved MC monolayer was observed in MS-275 treated mice. Representative images of peritoneal serial sections of a mouse from the vehicle group show cytokeratin (CK) and α-SMA staining overlapping in the sub-mesothelial compact zone. Immunohistochemical analysis shows CK expression limited to the preserved mesothelium of a mouse treated with MS-275. Scale bar: 50 μm. CAFs: Carcinoma-associated fibroblasts. MCs: Mesothelial cells. **E** Representative images of parietal peritoneal tissues show decreased sub-mesothelial FN-1 staining in an MS-275 treated mouse (right) as compared to a control (left). Scale bar: 50 𝜇m. Right panel shows the quantification of FN-1 staining (right) (*p < 0.05)

We next aimed to extend the analysis to a mouse model of EOC peritoneal carcinomatosis. Mice were treated with MS-275 and intraperitoneally inoculated with SKOV3-luc-D3 ovarian cancer cells. The experimental plan is described in Fig. 8A. Tumor-produced bioluminescence signal was monitored twice weekly for 40 days (Fig. 8B). Mouse weights were maintained during the experiment (Suppl. Fig. S10), indicating that MS-275, at the concentrations used, was well tolerated.

As shown in Fig. 8C, tumor metastatic implants visualized by visceral and peritoneal bioluminescence were significantly reduced in mice treated with MS-275 as compared with controls. Interestingly, treatment with MS-275 markedly reduced mesenchymal-like MC invading the submesothelial stroma, a process known to favor EOC metastasis (Fig. 8D). As a link with the in vitro investigation, we found markedly reduced FN-1 expression in the sub-mesothelial stroma in MS-275-treated mice (Fig. 8E).

Taken together, our results indicate that MS-275-mediated HDAC1–2 inhibition impairs EOC cell adhesiveness to MC, positioning it as a promising approach to tackle the first and crucial step of EOC peritoneal metastasis.

## Discussion

In this study, we describe the role of HDAC1/2 in promoting one of the first and crucial steps of EOC trans–coelomic metastasis (i.e. the adhesion of EOC cells to fibrotic peritoneum) and shed light on molecular mechanisms conferring MC permissiveness to EOC progression. This study demonstrates that adhesion of both EOC platinum-resistant and -sensitive cell lines to fibrotic MCs is markedly impaired by treatment with MS-275 at a concentration (250 nM) not affecting MC viability while enhancing histone H3 acetylation [24]. MS-275 is an inhibitor of all three HDAC1, HDAC2 and HDAC3 isoforms at nanomolar concentrations [23, 42].

Static adhesion experiments were complemented by peritoneal clearance assays, a more dynamic assay to analyze the complexity of MC/EOC interactions [38].

HDAC1–2 genetic silencing confirmed the role of these isoforms in the regulation of adhesion, whereas an implication of HDAC3 was ruled out using both a specific pharmacological inhibitor and siRNA-mediated silencing, although its specific role in the metastatic process cannot be excluded [43].

Experiments using blocking antibodies demonstrated that MCs/EOC cell adhesion is α5β1 integrin- and FN-1-dependent. Interestingly, the increased inhibition of adhesion observed by orthogonal treatment suggests that both MC and EOC integrins are implicated in this process. The observation of a major role of α5β1 integrin in mediating MC/EOC adhesion confirms previous studies in murine and human experimental systems [10, 34, 40].

When analyzing the mechanistic role of HDAC1–2 inhibition on MC/EOC adhesion, we found by RT-PCR and quantitative proteomic analysis that the expression of α5 and β1 integrin subunits is maintained or increased in MCs upon exposure to MS-275. Thus, inhibition of MC/EOC adhesion could not be explained by a downregulation of α5 and β1 integrin subunits. These data are in accordance with previous studies demonstrating that treatment with the pan-HDAC inhibitor trichostatin A promoted the induction of α4, β2 and β6 Integrin subunits in a hepatocyte cell line [44].

Using cytofluorimetric analysis and immunofluorescence and using two specific monoclonal antibodies, we demonstrated that treatment with MS-275 inhibited β1 integrin activity. To our knowledge, this is the first report linking HDAC1–2 inhibition to this process.

Integrin activity is critical for cell adhesion to ECM and is regulated by signals emerging from both the extracellular ligand (outside-in regulation) and the cytoplasm (inside-out regulation) [45].

Regarding outside-in regulation, ECM binding induces conformational changes in Integrins allowing the intracellular tails of the β subunits to interact with intracellular signaling and cytoskeletal molecules such as other Integrin subunits, Paxillin, Vinculin, Talin, FAK, Src, VASP, α-Actinin-1 and Zyxin [46].

As demonstrated by proteomic analysis, treatment with MS-275 led to profound changes in the expression of actin regulators. In particular, the expression of Talin-1, Zyxin, and α-Actinin-1 was altered after treatment with MS-275.

We focused on Talin-1, which is mostly localized at sites of cell-ECM linkage, where it plays a key role in integrin activation. Talin has β1-Integrin- and Actin-binding sites [47] and acts as a scaffold for the building of Actin cytoskeleton/Integrin/FN-1 connections [48]. Therefore, it is the main link between the Integrin cytoplasmatic tail and Actin fibers [49]. Talin-1 plays a major role in mesothelial clearance [38]. Interestingly, Talin-1 expression was increased in MCs surrounding tumor lesions in the peritoneum of EOC patients. As expected, Talin-1 silencing significantly inhibited both MC/EOC adhesion and β1 Integrin activity.

To further analyze the molecular mechanisms underlying, we focused on FN-1, the main α5β1 ligand. FN-1 expression by MCs is critical in MC/EOC interactions and is stimulated by EOC [40].

While RT-PCR and quantitative proteomic demonstrated that total levels of FN-1 were increased by treatment with MS-275, by WB analysis of intracellular and extracellular FN-1 and by immunofluorescence of decellularized matrices, we found that MS-275 impairs FN-1 secretion and organization. We proposed a causal relationship between decreased FN-1 secretion, aberrant fiber organization, and reduced adhesion, and we postulated that HDAC1/2 inhibition may play a part in this process.

After its release as a dimer, FN-1 undergoes integrin-mediated fibrillogenesis under the control of integrin activation to generate a meshwork of intertwined fibrils [50]. α5β1 integrin activation favors the recruitment of additional FN-1 molecules, promoting the organization of the FN-1 fibrillar network [51]. The role of HDAC inhibition in the regulation of FN-1 secretion has been scarcely analyzed so far.

In contrast with our results, Scriptaid, a selective inhibitor of HDACs 1/3/8, was demonstrated to inhibit transcriptionally the expression of FN-1 and type I Collagen in TGF-β1-treated murine and human CAFs [52]. To dissect the effect of MCs from that of the EOC counterpart is remarkably challenging. Especially in vivo. In line with previous results. we found that MS-275 modulates spheroid formation and growth [53, 54]. Defective β1 integrin activation may be implicated in a defective spheroid assembly, whereas a block of cell cycle progression may be linked to induction of p21 by MS-275 [55].

**Fig. 9:**
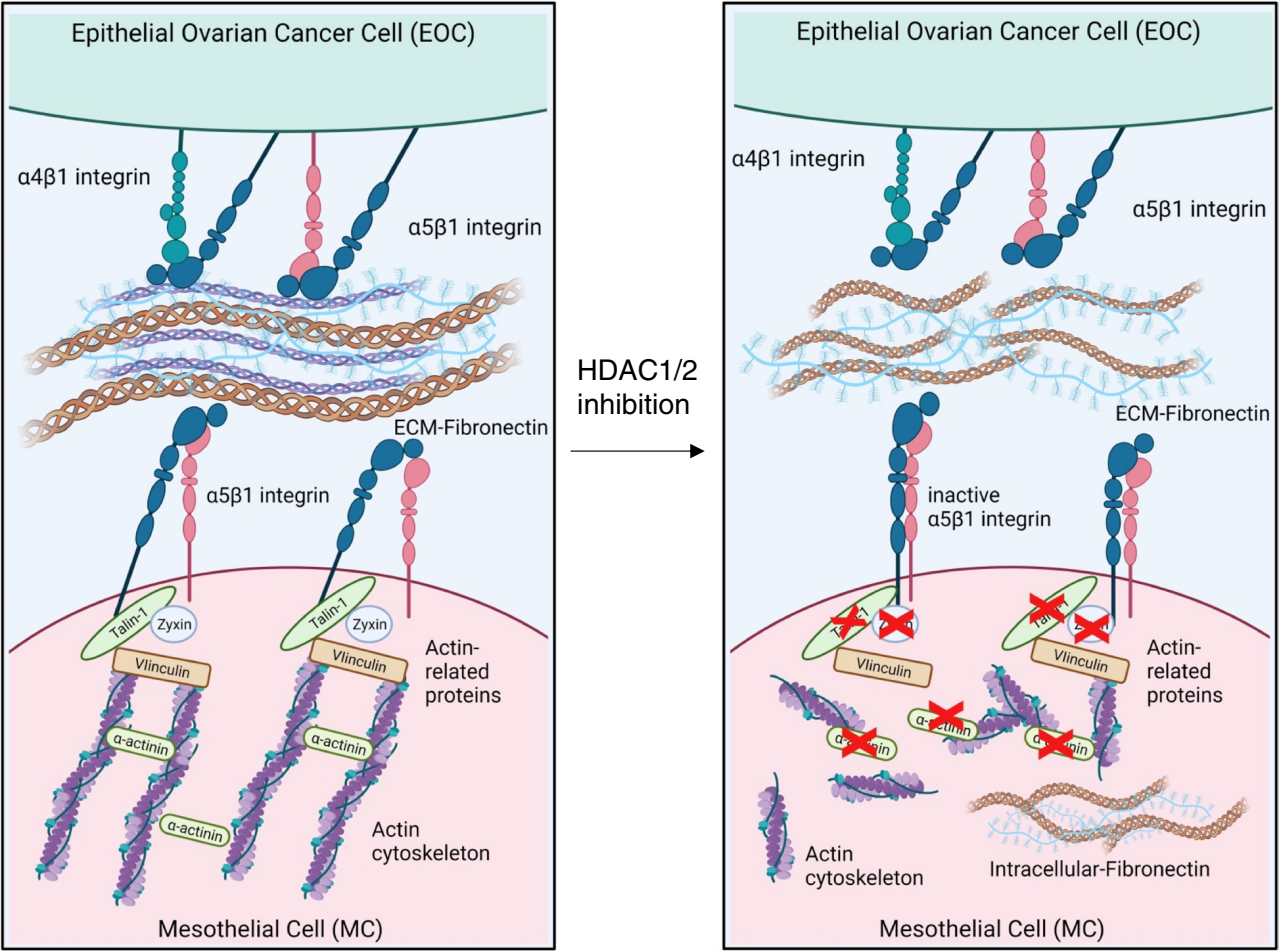
Schematic representation of the effect of HDAC1/2 inhibition on MC/EOC adhesion. HDAC1/2 inhibition downregulates the expression of Talin-1 and other cytoskeletal regulators. This alters the Actin network leading to α5β1 Integrin inactivation and impairing FN-1 secretion and eventually inhibiting MC/EOC adhesion

Overall, these data led us to elaborate a working model where HDAC1/2 inhibition causes: *i*) Downregulation of Talin-1 and other Actin-related proteins; ii) the decrease of β1 integrin activation; iii) inhibition of FN-1 deposition and organization within the ECM. This molecular mechanism is summarized in Fig. 9. Indeed, Talin-1 genetic silencing is sufficient to alter Actin polymerization, to inhibit MCs/EOC cell adhesion, as well as to create a pattern of FN-1 expression in decellularized matrices similar to that observed upon MS-275 treatment. Importantly, Talin-1 ectopic expression significantly rescued MC/EOC adhesion in MS-275 treated MCs.

This in vitro evidence was confirmed by an in vivo metastasis assay. MS-275 is an oral bioavailable drug with a half-life ranging from around 33–150 h [56]. The role of class I HDAC-specific inhibitors in cancer and especially in EOC therapy has already been analyzed in other studies. Treatment with MS-275 has been demonstrated to restore the epithelial differentiation in EOC and to abolish anchorage-independent growth in vitro [53]. The activity of class I HDAC inhibitors has been linked to immunomodulatory effects. In particular, MS-275 promotes the activation of intra-tumoral CD8 T cells [57, 58]. Our results demonstrate that HDAC1–2 inhibition by MS-275 may directly impact on the plasticity and functions of the MCs monolayer independently of the concomitant effects on EOC cells and on the regulation of the immune system.

Overall, this study elucidating the epigenetic regulation of a specific effect of MCs in the first crucial adhesion step of EOC metastasis enlightens a fundamental molecular mechanism in tumor transformation and may constitute the rationale for further translational studies.

### Supplementary Information

**Additional file 1.**

## Abbreviations

MMT: Mesothelial to Mesenchymal transition
EMT: Epithelial to Mesenchymal transition
EOC: Epithelial Ovarian Cancer
MC: Mesothelial Cell
HAT: Histone Acetyltransferases
HDAC: Histone Deacetylases
PD: Peritoneal Dialysis
TGFβ: Transforming Growth Factor-β
IL1β: Interleukin-1β
PDPN: Podoplanin
FAP: Fibroblast Activation Protein
FN-1: Fibronectin-1
ECM: Extracellular Matrix
CAF: Carcinoma-Associated Fibroblast
