# Supplementary material for: HDAC1/2 control mesothelium/ovarian cancer adhesive interactions impacting on Talin-1-α5β1-integrin-mediated actin cytoskeleton and extracellular matrix protein remodeling
Source: J Exp Clin Cancer Res. 2024 Jan 23;43:27. doi: 10.1186/s13046-023-02930-8 (PMC10804625; doi:10.1186/s13046-023-02930-8)
Supplement: Supplementary file 1 — Additional file 1. [file 13046_2023_2930_MOESM1_ESM.zip › 13046_2023_2930_MOESM1_ESM.docx]

**SUPPLEMENTARY FIGURE LEGENDS**

**Figure S1: Treatment of MeT5A cells with TGFβ in combination with IL-1β increases SKOV3/MeT5A adhesion.**

(**A**) Representative images and quantifications of GFP-labelled SKOV3 cells adhering to MeT5A cells left untreated or treated with TGFβ1 (2ng/ml) in combination with IL-1β (0.5 ng/ml), indicated as (T+I), for 48h. Nuclei are stained with DAPI (blue). Results are shown as relative number of adherent SKOV3 cells. 3 fields for each sample were analyzed. This experiment was performed three times in triplicate. Scale bar: 25 μm. Differences were considered significant at P<0.05 (**p* < 0.05; ***p* < 0.01; ****p* < 0.001). (**B-C**) Representative images and quantifications of GFP-labelled SKOV3 (**B**) and OVCAR3 cells (**C**) adhering to MeT5A. When reported, MeT5A cells were treated with MS-275 (250 nM) SKOV3 cells were treated with MS-275 (2.5 μM) OVCAR cells were treated with MS-275 (1 μM) for 72 h before performing the adhesion experiment. Results are shown as relative number of adherent SKOV3 cells. 3 fields for each sample were analyzed. Each experiment was performed at least 3 times in triplicate. Scale bar: 25 μm. Differences were considered significant at P<0.05 (**p* < 0.05; ***p* < 0.01; ****p* < 0.001).

**Figure S2: mesenchymal-like MeT5A/SKOV3 adhesion is independent of HDAC3 inhibition.** (**A**) Representative images showing GFP-labelled SKOV3 cells adhering to MeT5A cell monolayers after pre-treatment with MC-3105 (250nM), an HDAC3 selective inhibitor. (**B**) qRT-PCR showing genetic silencing of HDAC3 from total RNA of MeT5A cells used for the experiment shown in (**C).** Bars represent means±SEM of 3 experiments (**C**) Representative images showing GFP-labelled SKOV3 cells adhering to MeT5A cell monolayers after HDAC3 silencing. Nuclei are stained with DAPI (blue). Results are shown as relative number of adherent EOC. Adherent SKOV3 cells were evaluated in 3 fields/sample. Each experiment was performed at least 3 times in triplicate. Scale bar: 25 μm. Differences were considered significant at P<0.05 (**p* < 0.05; ***p* < 0.01; ****p* < 0.001).

**Figure S3: Treatment with MS-275 modifies the proteome of primary mesothelial cells**. Primary MCs were left untreated or treated for 72 hours with MS-275 (250 nM) (N=3), cells were lysed with RIPA buffer and quantified by Bradford assay. Total lysates were digested and separated in 8 fractions based on proteins hydrophobic properties. Separated fractions were analysed by label-free liquid chromatography-mass spectrometry (nLC-MS/MS). (**A**) Principal component analysis (PCA) of the LFQ intensities obtained in NT and MS-275 treated sample datasets. (**B**) Volcano plots comparing NT (left panel) and MS-275 (right panel) upregulated proteins. Black curves represent the significance threshold at false discovery rate (FDR) of 0.05 and S0 of 0.1. (**C**) Heat map of differentially expressed proteins in NT and MS-275 samples. LFQ intensities were expressed in z-score values (range of intensity z-score: ±2.5). Up-regulated and down-regulated proteins are expressed in red and green scale respectively. Hierarchical clustering was performed using Euclidean distance and average linkage using the Perseus software. (**D**) Table showing selected identified proteins belonging to specific Gene ontology biological processes (GOBP) shown in the right column. (**E**) Gene Ontology enrichment analysis performed by Perseus software on differentially expressed proteins between NT and MS-275 datasets. GOBP: gene ontology biological processes; GOCC: Gene ontology cellular components; GOMF: Gene ontology molecular functions.

**Figure S4: Comparative analysis between MeT5A cells and primary MCs proteomic datasets.** (**A-B**) Pearson correlation test performed between Met5A and primary MCs LFQ intensities in CTR and MS-275 treated samples (Perseus software). Differences were considered significant at P<0.05. (**C-D**) Venn diagrams of MS-275 modulated proteins in Met5A cells intersecting with primary MCS-related modulated proteins. LFQ: label free quantification; r: Pearson correlation coefficient.

**Figure S5: List of upregulated protein in MS-275 treated sample common to primary MCs and Met5A cells identified by quantitative mass spectrometry analysis.**

**Figure S6: List of downregulated protein in MS-275 treated sample common to primary MCs and Met5A cells identified by quantitative mass spectrometry analysis.**

**Figure S7: Effects of MS-275 on β1 Integrin activity in primary MCs.** Immunofluorescence showing primary MCs treated with MS-275 (250 nM) for 72 hours. Fixed cells were stained with an antibody against total β1 Integrins or against active β1 Integrins (9EG7). The quantification of the experiment is shown on the right. Mander’s colocalization M2 coefficients were measured using the JACoP plugin on ImageJ. At least 6 images were quantified per experiment. Confocal images are shown from one representative experiment of three performed. Differences were considered significant at P<0.05 (**p* < 0.05; ***p* < 0.01; ****p* < 0.001; **** *p* < 0.0001).

**Figure S8:** **HDAC1/2 genetic silencing impact on FN-1 and actin remodellers expression**. (**A**) RT-qPCR experiments show the expression of FN-1, Talin-1, Zyxin**,** and α-Actinin-1 from total RNA of mesenchymal-like MeT5A after HDAC1, HDAC2 and both HDAC1-2 genetic silencing. Bars represent means±SEM of three experiments. Differences were considered significant at P<0.05 (**p* < 0.05; ***p* < 0.01; ****p* < 0.001; **** *p* < 0.0001).(**B**) Representative Western blot showing expression of HDAC1, HDAC2, FN-1 and Talin-1 from cell lysates of mesenchymal-like MeT5A cells treated as above. HSP90 was used as a loading control.

**Figure S9: Treatment with MS-275 downregulates the expression of Actin remodellers in MeT5A cells (A)** RT-qPCR experiments showing the expression of Talin-1 **(A)**, Zyxin **(B),** and α-Actinin-1 **(C)** from total RNA of mesenchymal-like MeT5A cells treated with MS-275 (250 nM) for 72 h. Bars represent means±SEM of three experiments. Differences were considered significant at P<0.05 (**p* < 0.05; ***p* < 0.01; ****p* < 0.001; **** *p* < 0.0001).

**Figure S10: MS-275 treatment does not cause significative weight changes in mice.** Histogram showing the weight of control and MS-275-treated mice during the in vivo experiment. Mice were weighted every week. No significant differences were found among the two groups of mice during the study.
