# Supplementary material for: HDAC1/2 control mesothelium/ovarian cancer adhesive interactions impacting on Talin-1-α5β1-integrin-mediated actin cytoskeleton and extracellular matrix protein remodeling
Source: J Exp Clin Cancer Res. 2024 Jan 23;43:27. doi: 10.1186/s13046-023-02930-8 (PMC10804625; doi:10.1186/s13046-023-02930-8)
Supplement: Supplementary file 1 — Additional file 1. [file 13046_2023_2930_MOESM1_ESM.zip › Supplementary Figures - .pptx]

## Slide 1
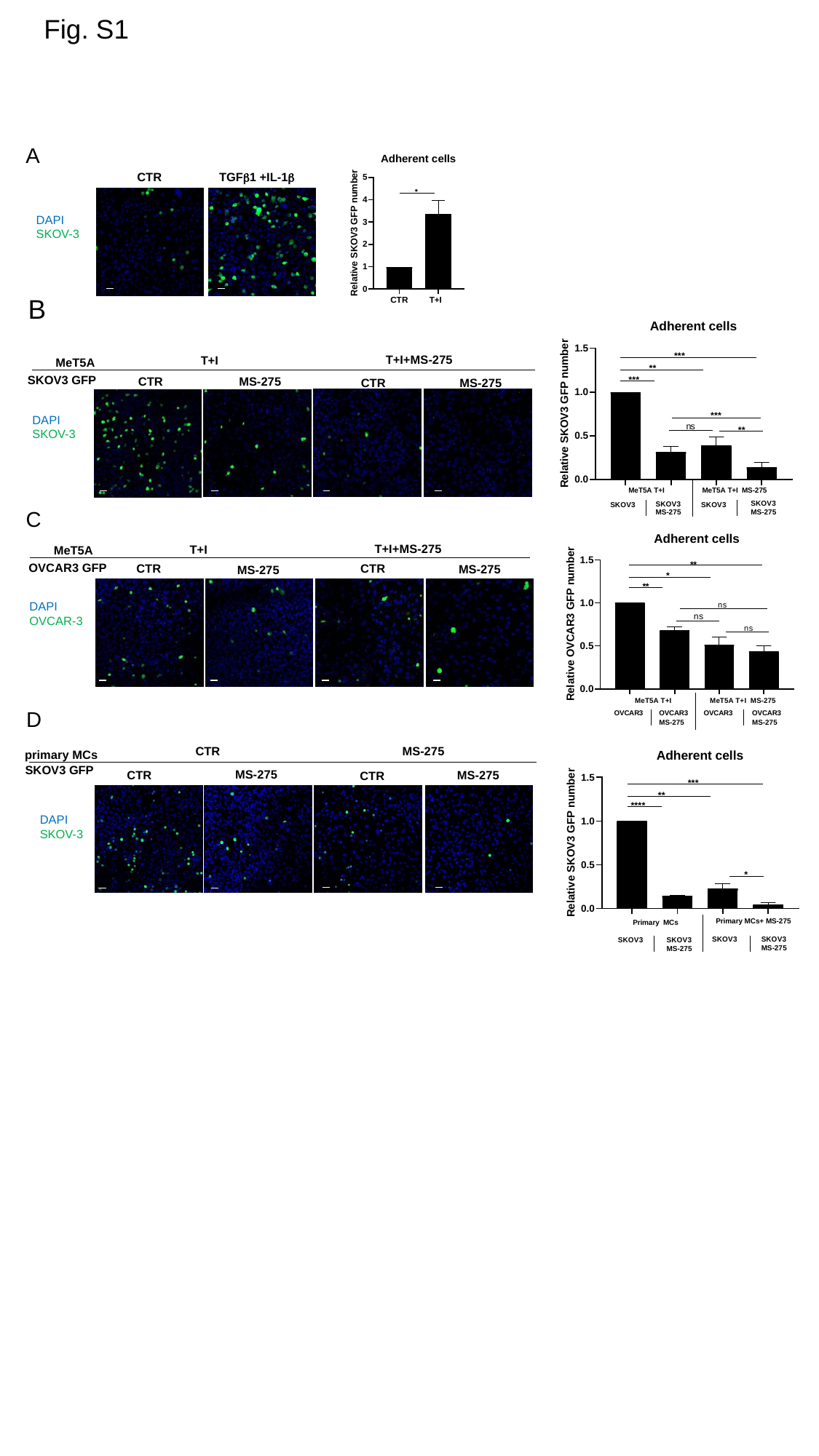

Fig. S1
A
TGFb1 +IL-1b
CTR
DAPI
SKOV-3
B
 T+I+MS-275
T+I
MeT5A
 MS-275
CTR
SKOV3 GFP
CTR
 MS-275
DAPI
SKOV-3
C
 T+I+MS-275
T+I
MeT5A
 MS-275
OVCAR3 GFP
CTR
CTR
 MS-275
DAPI
OVCAR-3
D
CTR
 MS-275
primary MCs
SKOV3 GFP
MS-275
 MS-275
CTR
CTR
DAPI
SKOV-3

## Slide 2
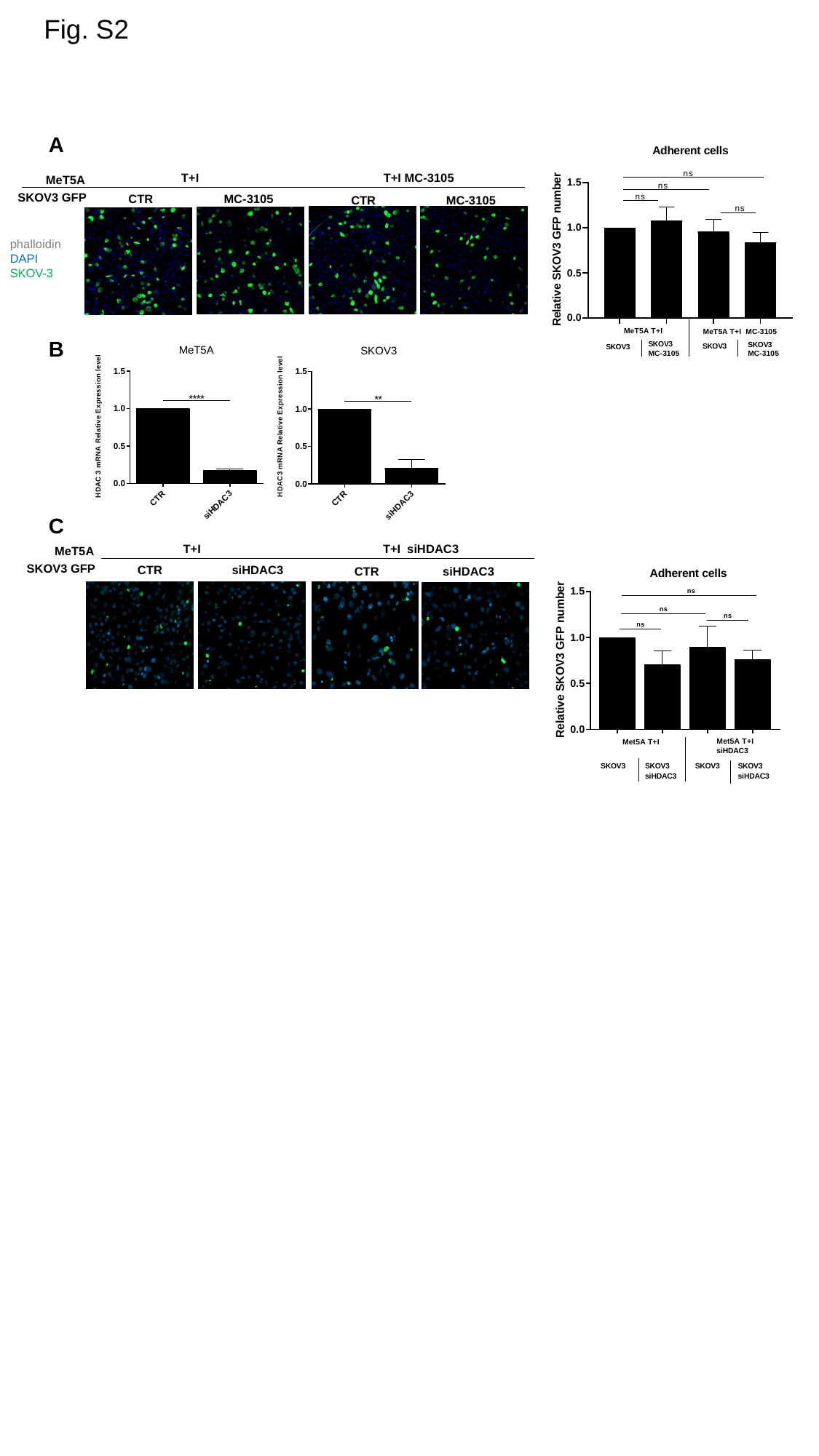

Fig. S2
A
T+I MC-3105
T+I
MeT5A
 MC-3105
CTR
SKOV3 GFP
CTR
 MC-3105
phalloidin
DAPI
SKOV-3
B
C
T+I siHDAC3
T+I
MeT5A
 siHDAC3
CTR
SKOV3 GFP
CTR
 siHDAC3

## Slide 3
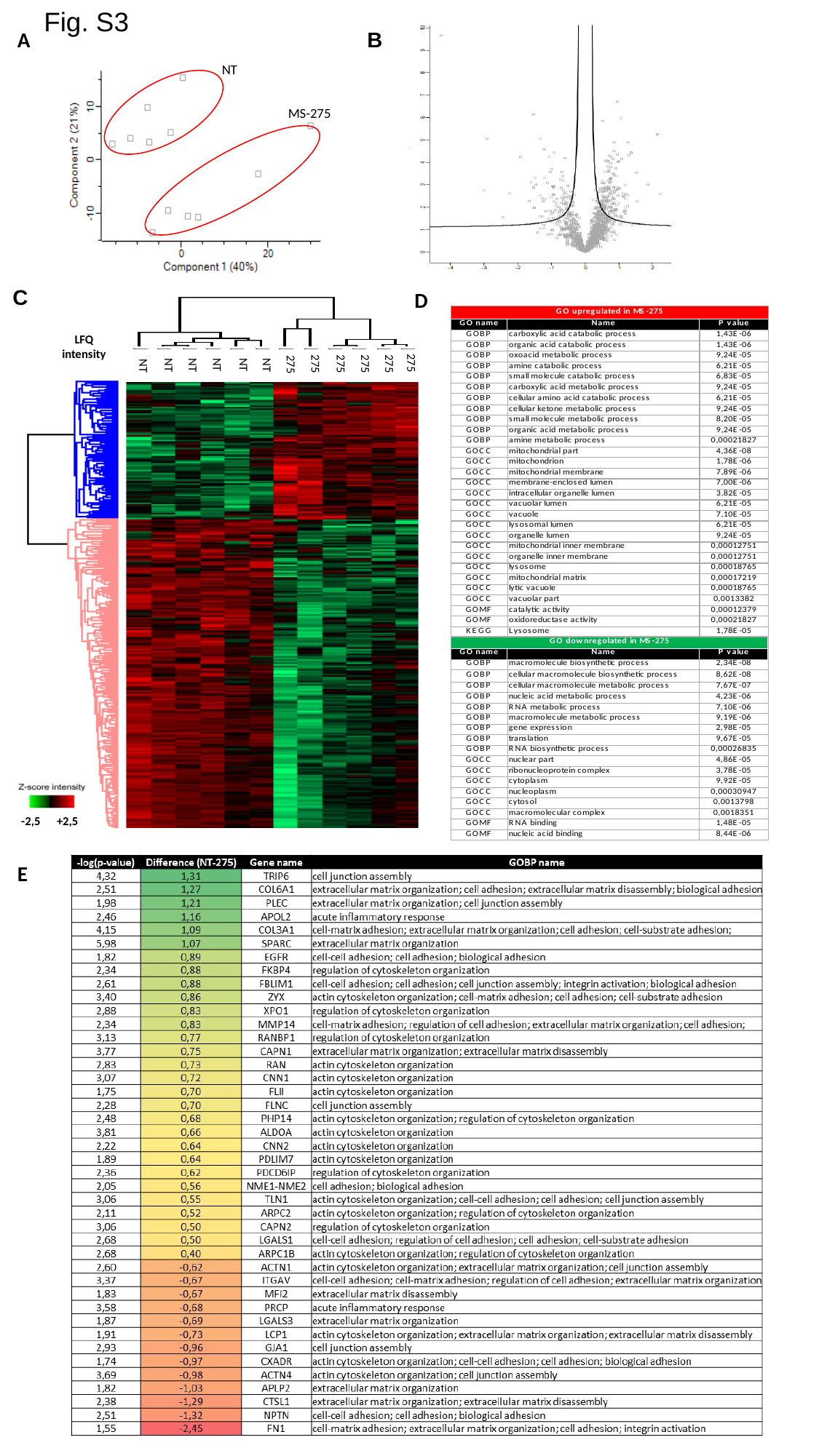

Fig. S3
A
B
NT
MS-275
C
D
LFQ
intensity
275
275
275
275
275
275
NT
NT
NT
NT
NT
NT
-2,5 +2,5
E

## Slide 4
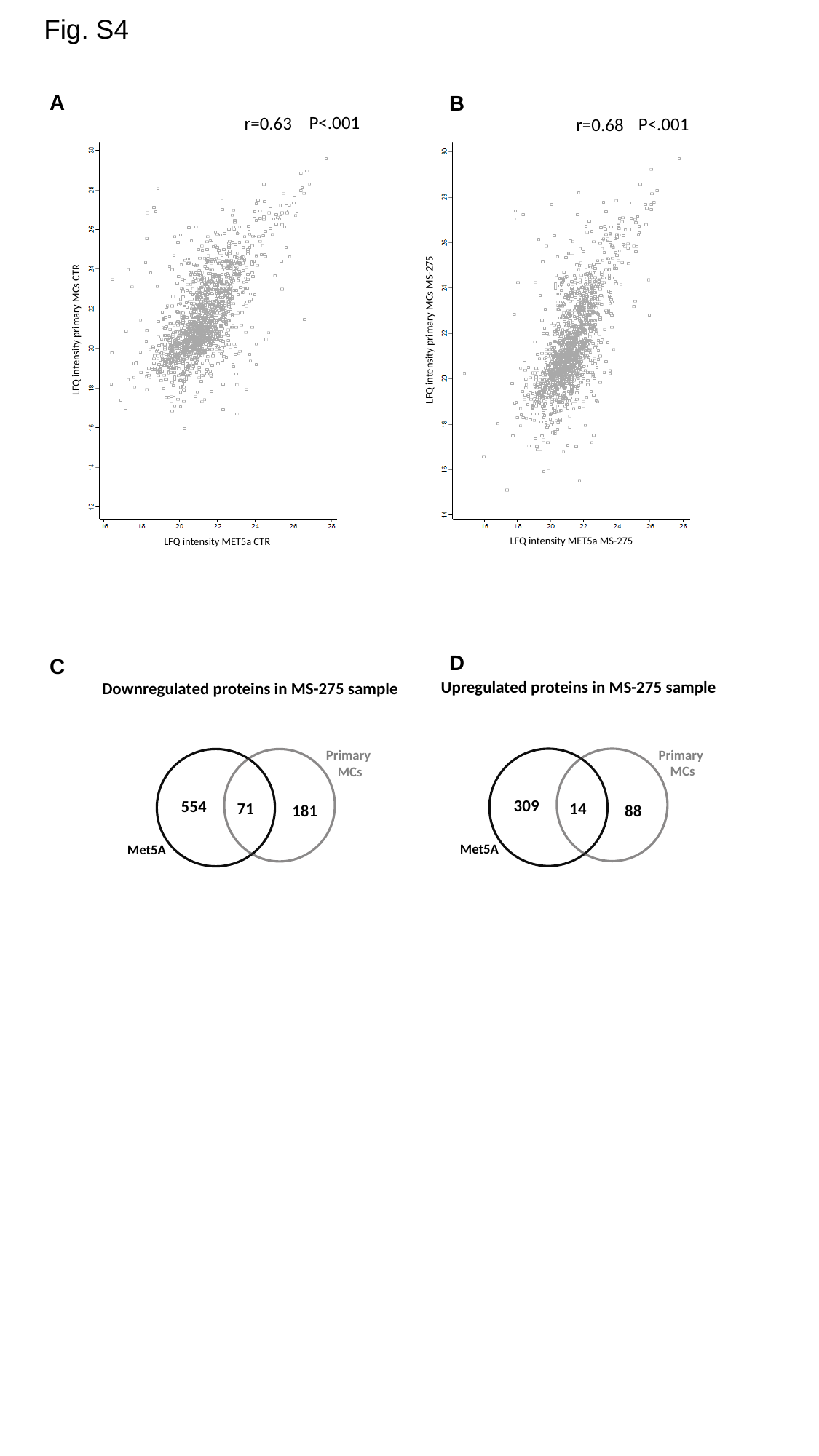

Fig. S4
A
B
P<.001
r=0.63
Ρ<.001
r=0.68
LFQ intensity primary MCs MS-275
LFQ intensity MET5a MS-275
LFQ intensity primary MCs CTR
LFQ intensity MET5a CTR
D
C
Upregulated proteins in MS-275 sample
Downregulated proteins in MS-275 sample
Primary
MCs
Primary
MCs
554
71
181
Met5A
309
14
88
Met5A

## Slide 5
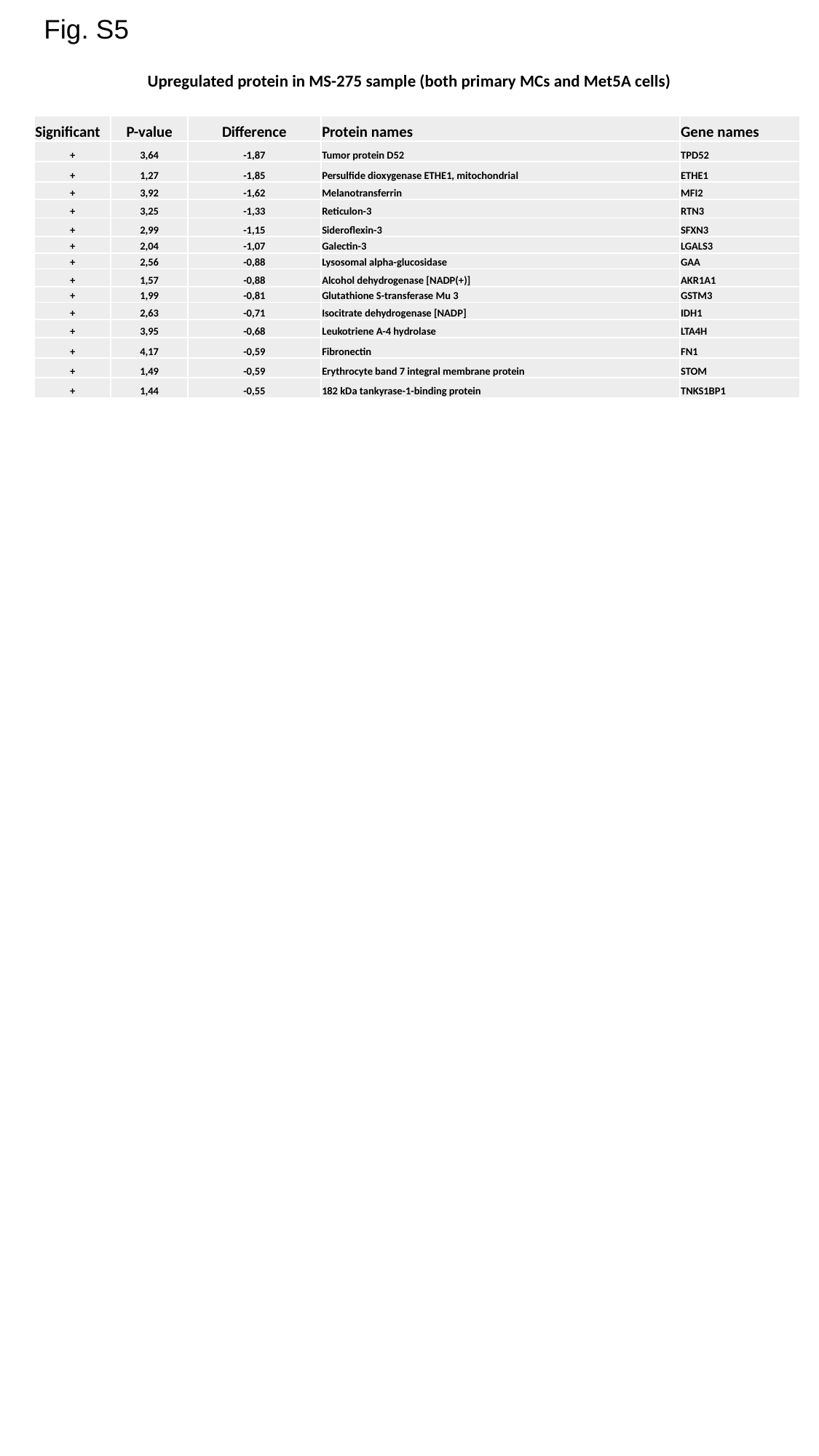

Fig. S5
Upregulated protein in MS-275 sample (both primary MCs and Met5A cells)
| Significant | P-value | Difference | Protein names | Gene names |
| --- | --- | --- | --- | --- |
| + | 3,64 | -1,87 | Tumor protein D52 | TPD52 |
| + | 1,27 | -1,85 | Persulfide dioxygenase ETHE1, mitochondrial | ETHE1 |
| + | 3,92 | -1,62 | Melanotransferrin | MFI2 |
| + | 3,25 | -1,33 | Reticulon-3 | RTN3 |
| + | 2,99 | -1,15 | Sideroflexin-3 | SFXN3 |
| + | 2,04 | -1,07 | Galectin-3 | LGALS3 |
| + | 2,56 | -0,88 | Lysosomal alpha-glucosidase | GAA |
| + | 1,57 | -0,88 | Alcohol dehydrogenase [NADP(+)] | AKR1A1 |
| + | 1,99 | -0,81 | Glutathione S-transferase Mu 3 | GSTM3 |
| + | 2,63 | -0,71 | Isocitrate dehydrogenase [NADP] | IDH1 |
| + | 3,95 | -0,68 | Leukotriene A-4 hydrolase | LTA4H |
| + | 4,17 | -0,59 | Fibronectin | FN1 |
| + | 1,49 | -0,59 | Erythrocyte band 7 integral membrane protein | STOM |
| + | 1,44 | -0,55 | 182 kDa tankyrase-1-binding protein | TNKS1BP1 |

## Slide 6
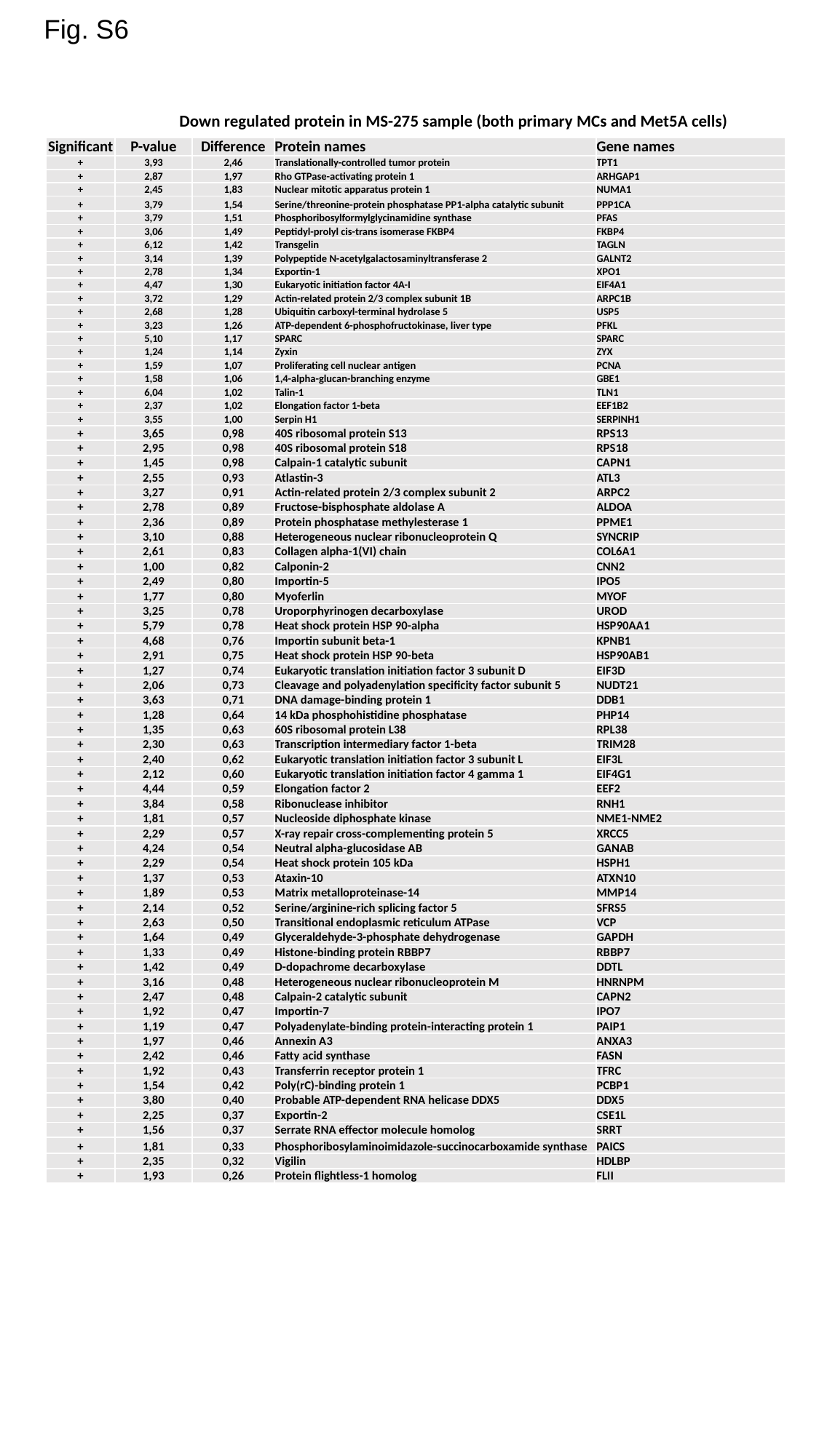

Fig. S6
Down regulated protein in MS-275 sample (both primary MCs and Met5A cells)
| Significant | P-value | Difference | Protein names | Gene names |
| --- | --- | --- | --- | --- |
| + | 3,93 | 2,46 | Translationally-controlled tumor protein | TPT1 |
| + | 2,87 | 1,97 | Rho GTPase-activating protein 1 | ARHGAP1 |
| + | 2,45 | 1,83 | Nuclear mitotic apparatus protein 1 | NUMA1 |
| + | 3,79 | 1,54 | Serine/threonine-protein phosphatase PP1-alpha catalytic subunit | PPP1CA |
| + | 3,79 | 1,51 | Phosphoribosylformylglycinamidine synthase | PFAS |
| + | 3,06 | 1,49 | Peptidyl-prolyl cis-trans isomerase FKBP4 | FKBP4 |
| + | 6,12 | 1,42 | Transgelin | TAGLN |
| + | 3,14 | 1,39 | Polypeptide N-acetylgalactosaminyltransferase 2 | GALNT2 |
| + | 2,78 | 1,34 | Exportin-1 | XPO1 |
| + | 4,47 | 1,30 | Eukaryotic initiation factor 4A-I | EIF4A1 |
| + | 3,72 | 1,29 | Actin-related protein 2/3 complex subunit 1B | ARPC1B |
| + | 2,68 | 1,28 | Ubiquitin carboxyl-terminal hydrolase 5 | USP5 |
| + | 3,23 | 1,26 | ATP-dependent 6-phosphofructokinase, liver type | PFKL |
| + | 5,10 | 1,17 | SPARC | SPARC |
| + | 1,24 | 1,14 | Zyxin | ZYX |
| + | 1,59 | 1,07 | Proliferating cell nuclear antigen | PCNA |
| + | 1,58 | 1,06 | 1,4-alpha-glucan-branching enzyme | GBE1 |
| + | 6,04 | 1,02 | Talin-1 | TLN1 |
| + | 2,37 | 1,02 | Elongation factor 1-beta | EEF1B2 |
| + | 3,55 | 1,00 | Serpin H1 | SERPINH1 |
| + | 3,65 | 0,98 | 40S ribosomal protein S13 | RPS13 |
| + | 2,95 | 0,98 | 40S ribosomal protein S18 | RPS18 |
| + | 1,45 | 0,98 | Calpain-1 catalytic subunit | CAPN1 |
| + | 2,55 | 0,93 | Atlastin-3 | ATL3 |
| + | 3,27 | 0,91 | Actin-related protein 2/3 complex subunit 2 | ARPC2 |
| + | 2,78 | 0,89 | Fructose-bisphosphate aldolase A | ALDOA |
| + | 2,36 | 0,89 | Protein phosphatase methylesterase 1 | PPME1 |
| + | 3,10 | 0,88 | Heterogeneous nuclear ribonucleoprotein Q | SYNCRIP |
| + | 2,61 | 0,83 | Collagen alpha-1(VI) chain | COL6A1 |
| + | 1,00 | 0,82 | Calponin-2 | CNN2 |
| + | 2,49 | 0,80 | Importin-5 | IPO5 |
| + | 1,77 | 0,80 | Myoferlin | MYOF |
| + | 3,25 | 0,78 | Uroporphyrinogen decarboxylase | UROD |
| + | 5,79 | 0,78 | Heat shock protein HSP 90-alpha | HSP90AA1 |
| + | 4,68 | 0,76 | Importin subunit beta-1 | KPNB1 |
| + | 2,91 | 0,75 | Heat shock protein HSP 90-beta | HSP90AB1 |
| + | 1,27 | 0,74 | Eukaryotic translation initiation factor 3 subunit D | EIF3D |
| + | 2,06 | 0,73 | Cleavage and polyadenylation specificity factor subunit 5 | NUDT21 |
| + | 3,63 | 0,71 | DNA damage-binding protein 1 | DDB1 |
| + | 1,28 | 0,64 | 14 kDa phosphohistidine phosphatase | PHP14 |
| + | 1,35 | 0,63 | 60S ribosomal protein L38 | RPL38 |
| + | 2,30 | 0,63 | Transcription intermediary factor 1-beta | TRIM28 |
| + | 2,40 | 0,62 | Eukaryotic translation initiation factor 3 subunit L | EIF3L |
| + | 2,12 | 0,60 | Eukaryotic translation initiation factor 4 gamma 1 | EIF4G1 |
| + | 4,44 | 0,59 | Elongation factor 2 | EEF2 |
| + | 3,84 | 0,58 | Ribonuclease inhibitor | RNH1 |
| + | 1,81 | 0,57 | Nucleoside diphosphate kinase | NME1-NME2 |
| + | 2,29 | 0,57 | X-ray repair cross-complementing protein 5 | XRCC5 |
| + | 4,24 | 0,54 | Neutral alpha-glucosidase AB | GANAB |
| + | 2,29 | 0,54 | Heat shock protein 105 kDa | HSPH1 |
| + | 1,37 | 0,53 | Ataxin-10 | ATXN10 |
| + | 1,89 | 0,53 | Matrix metalloproteinase-14 | MMP14 |
| + | 2,14 | 0,52 | Serine/arginine-rich splicing factor 5 | SFRS5 |
| + | 2,63 | 0,50 | Transitional endoplasmic reticulum ATPase | VCP |
| + | 1,64 | 0,49 | Glyceraldehyde-3-phosphate dehydrogenase | GAPDH |
| + | 1,33 | 0,49 | Histone-binding protein RBBP7 | RBBP7 |
| + | 1,42 | 0,49 | D-dopachrome decarboxylase | DDTL |
| + | 3,16 | 0,48 | Heterogeneous nuclear ribonucleoprotein M | HNRNPM |
| + | 2,47 | 0,48 | Calpain-2 catalytic subunit | CAPN2 |
| + | 1,92 | 0,47 | Importin-7 | IPO7 |
| + | 1,19 | 0,47 | Polyadenylate-binding protein-interacting protein 1 | PAIP1 |
| + | 1,97 | 0,46 | Annexin A3 | ANXA3 |
| + | 2,42 | 0,46 | Fatty acid synthase | FASN |
| + | 1,92 | 0,43 | Transferrin receptor protein 1 | TFRC |
| + | 1,54 | 0,42 | Poly(rC)-binding protein 1 | PCBP1 |
| + | 3,80 | 0,40 | Probable ATP-dependent RNA helicase DDX5 | DDX5 |
| + | 2,25 | 0,37 | Exportin-2 | CSE1L |
| + | 1,56 | 0,37 | Serrate RNA effector molecule homolog | SRRT |
| + | 1,81 | 0,33 | Phosphoribosylaminoimidazole-succinocarboxamide synthase | PAICS |
| + | 2,35 | 0,32 | Vigilin | HDLBP |
| + | 1,93 | 0,26 | Protein flightless-1 homolog | FLII |

## Slide 7
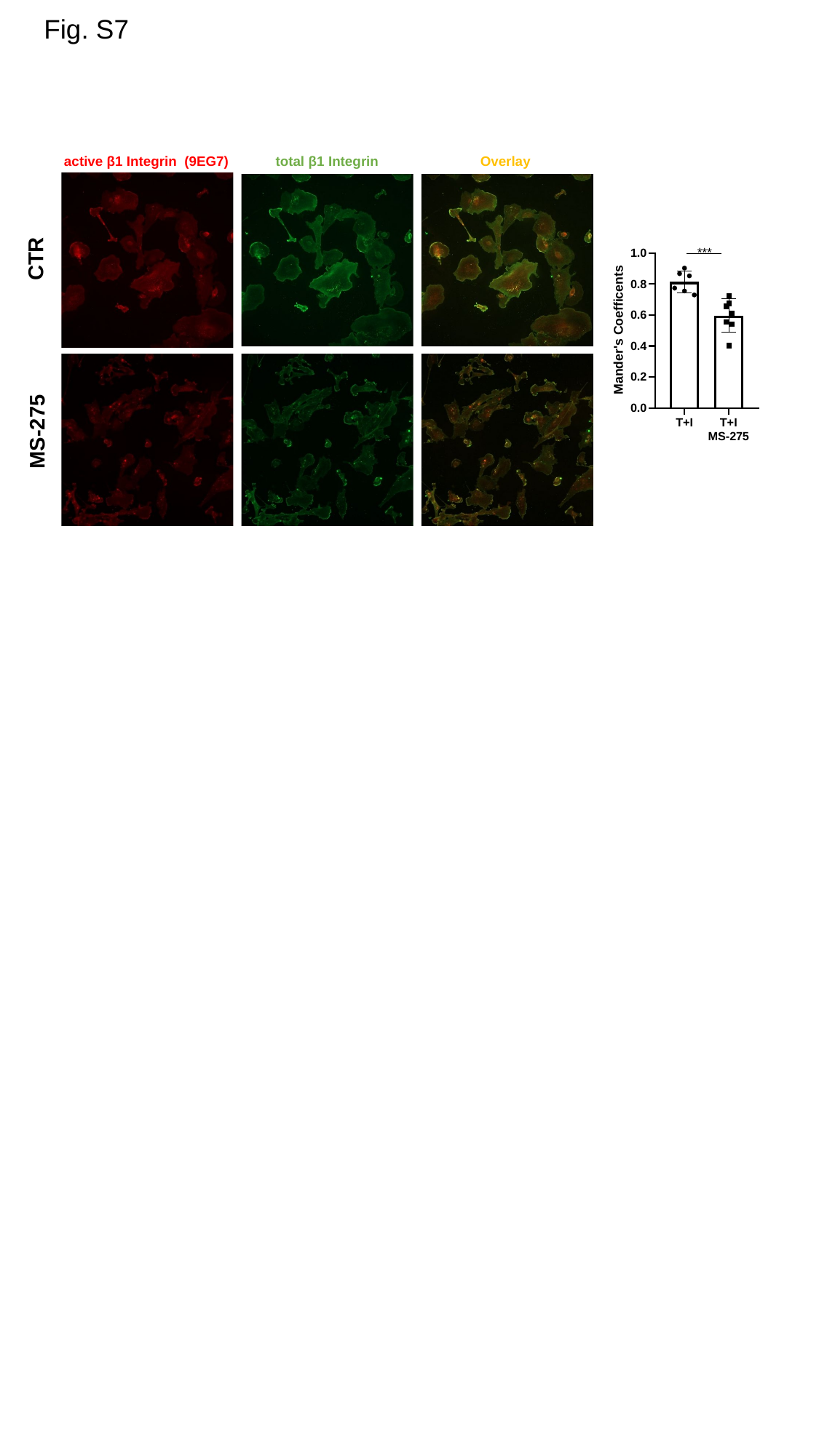

Fig. S7
active β1 Integrin (9EG7)
total β1 Integrin
Overlay
CTR
 MS-275

## Slide 8
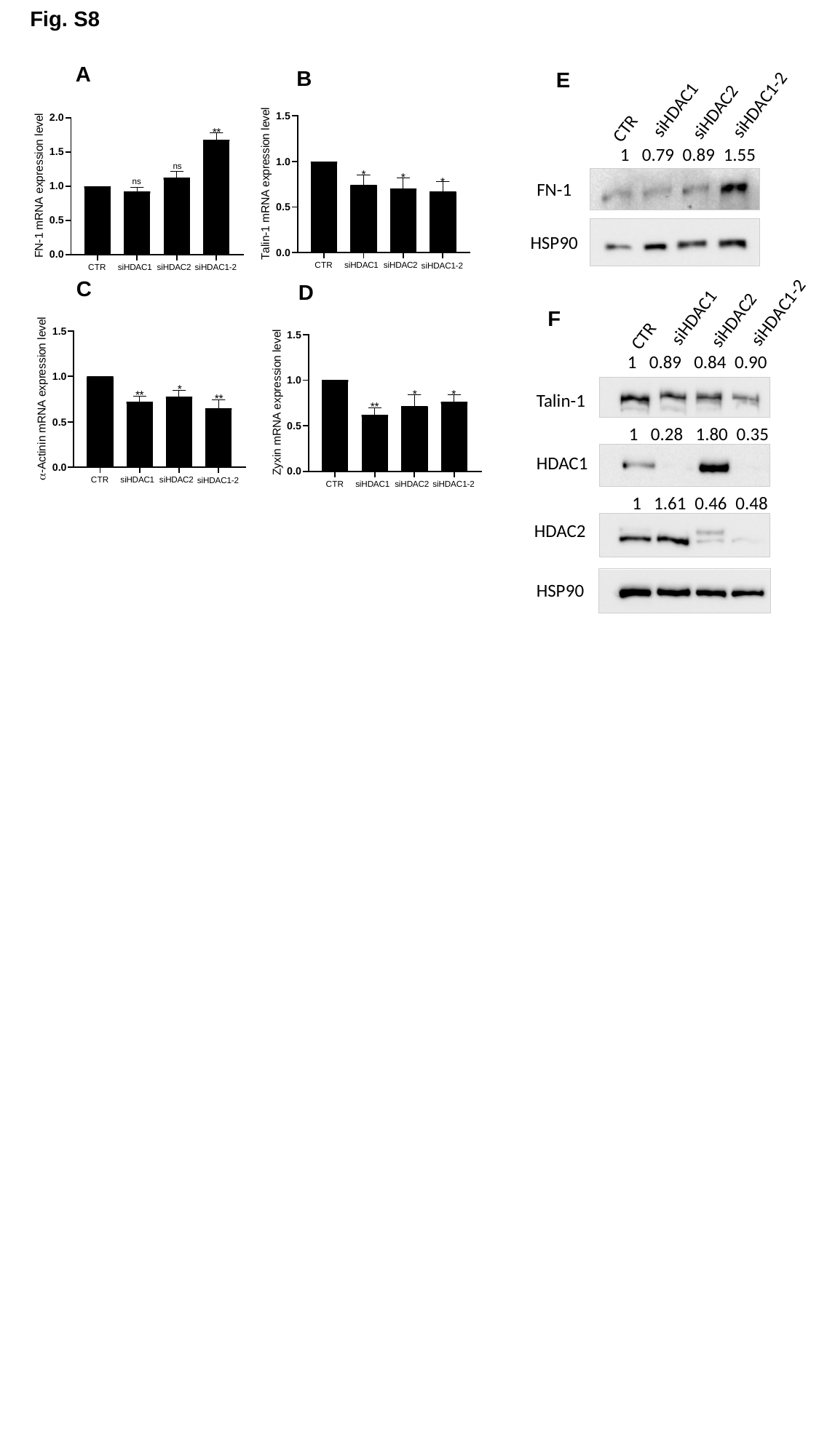

Fig. S8
A
B
E
siHDAC1
siHDAC1-2
siHDAC2
CTR
1 0.79 0.89 1.55
FN-1
HSP90
C
D
siHDAC1
siHDAC1-2
siHDAC2
CTR
F
1 0.89 0.84 0.90
Talin-1
1 0.28 1.80 0.35
HDAC1
1 1.61 0.46 0.48
HDAC2
HSP90

## Slide 9
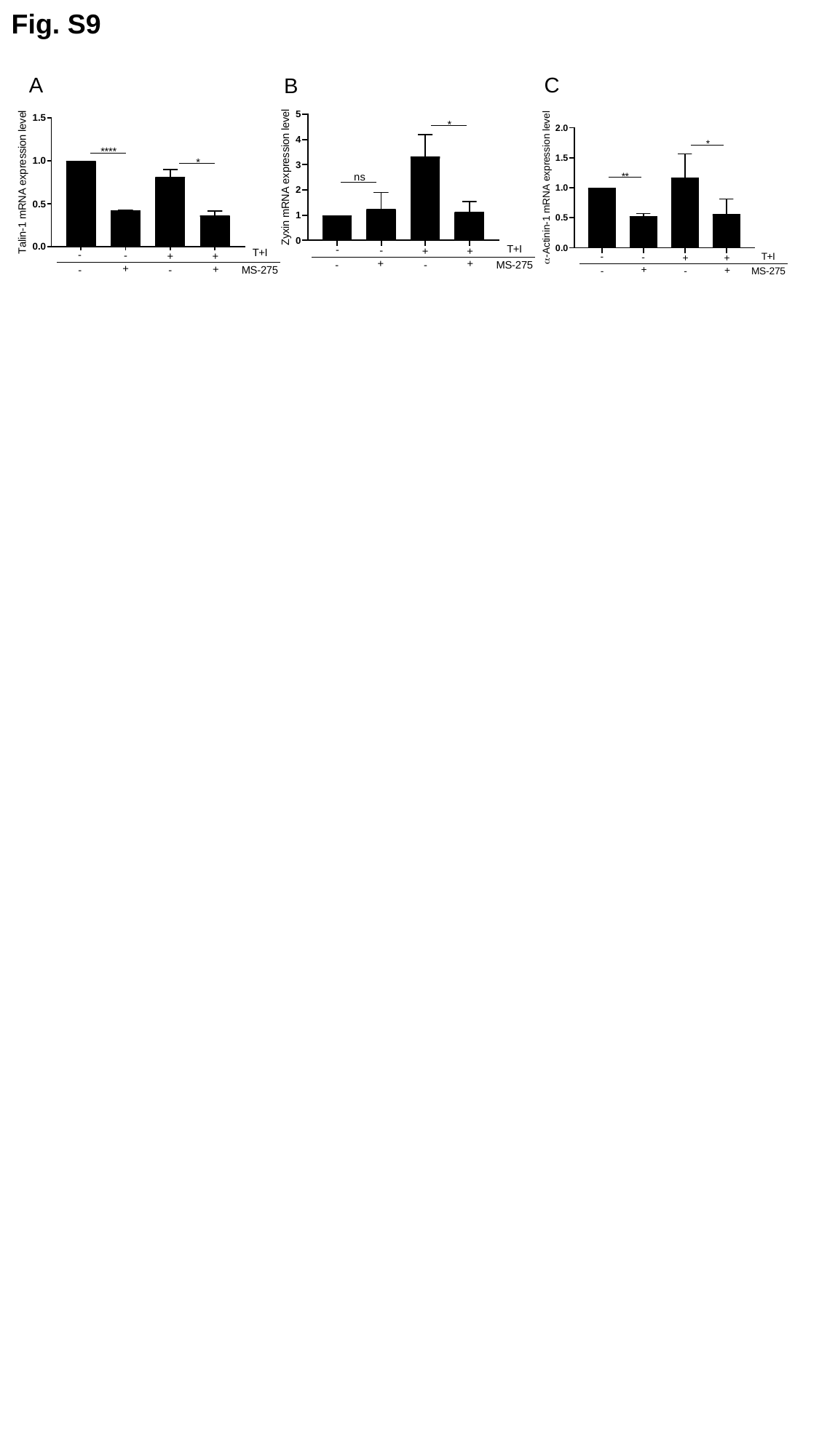

Fig. S9
A
C
B

## Slide 10
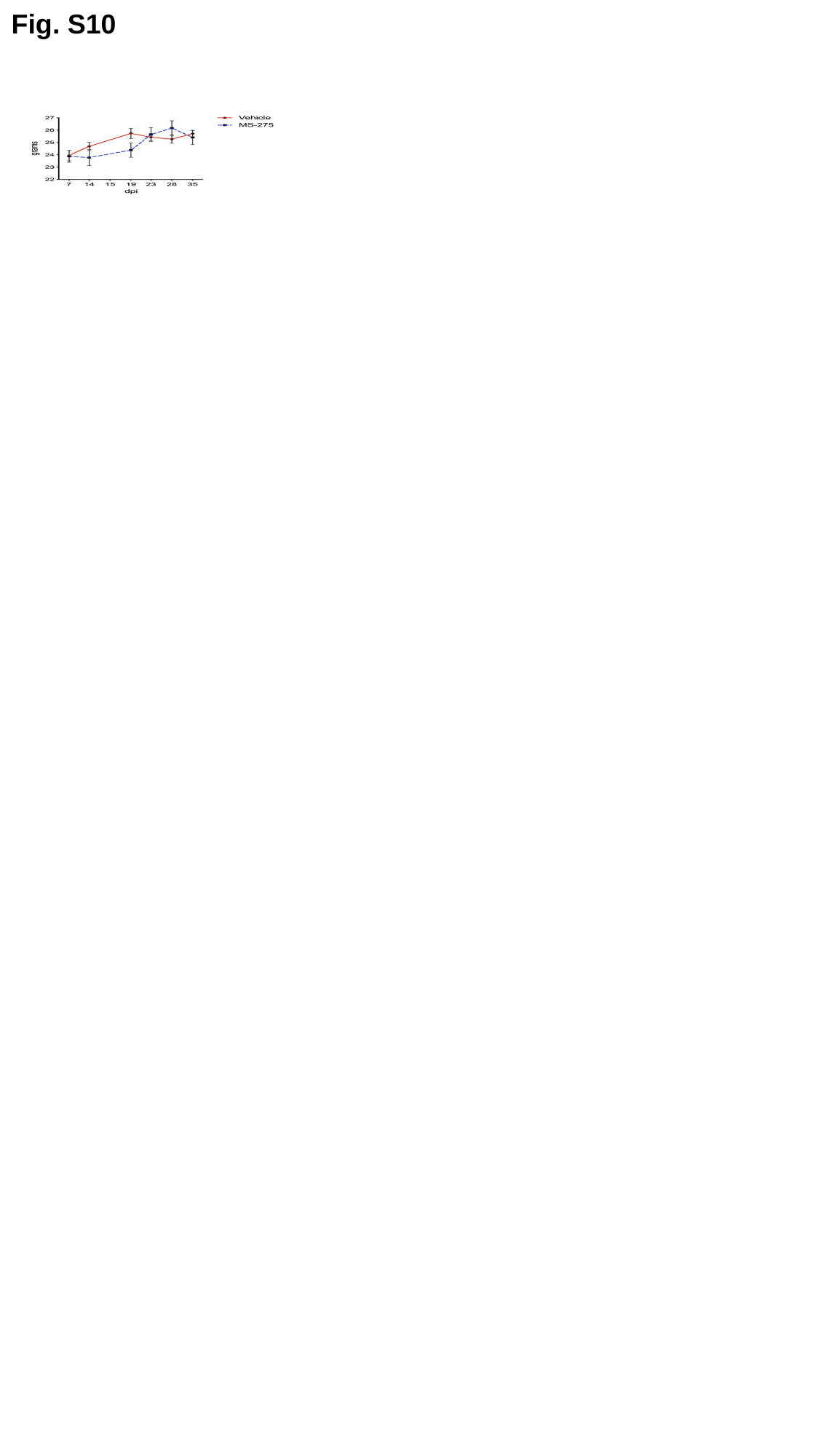

Fig. S10
